# Too much? Mortality and health service utilisation among Danish children 1999-2016: A register-based study

**DOI:** 10.1371/journal.pone.0224544

**Published:** 2019-10-30

**Authors:** Andreas Jensen, Per Kragh Andersen, John Sahl Andersen, Gorm Greisen, Lone Graff Stensballe

**Affiliations:** 1 Department of Paediatrics and Adolescent Medicine, Rigshospitalet, Copenhagen University Hospital, Copenhagen, Denmark; 2 Section of Biostatistics, Department of Public Health, University of Copenhagen, Copenhagen, Denmark; 3 Section of General Practice, Department of Public Health and Research Unit for General Practice, University of Copenhagen, Copenhagen, Denmark; 4 Department of Neonatology, Rigshospitalet, Copenhagen University Hospital and the University of Copenhagen, Copenhagen, Denmark; Yokohama City University, JAPAN

## Abstract

**Objectives:**

To describe the temporal development of mortality and health service utilisation defined as in- and outpatient hospital contacts, contacts with general practitioner and specialists, and prescribed dispensed medication among Danish children 0–5 years of age from 1999 to 2016.

**Design:**

Register-based descriptive study.

**Participants:**

All children born in Denmark in the period 1994–2016 followed until 5 years of age.

**Main outcome measures:**

Annual incidence rates of mortality and health service utilisation outcomes, and incidence rate ratios compared to the reference calendar year 1999. The new measure of post-discharge mortality is presented.

**Results:**

Post-discharge mortality decreased from 1999 to 2016, IRR_2016_ = 0.49 (95% CI: 0.36 to 0.66). Total contacts did not change much over time, IRR_2016_ = 1.02 (1.02 to 1.03), but increased among neonates, IRR_2016_ = 3.69 (3.63 to 3.75), and decreased among children with chronic disease IRR_2016_ = 0.94 (0.93 to 0.94). In- and out-patient hospitalisations increased, IRR_2016_ = 1.26 (1.24–1.27) resp. IRR_2016_ = 1.62 (1.60–1.63), contacts with medical specialists increased, IRR_2016_ = 1.43 (1.42 to 1.43), whilst contacts with general practitioner decreased, IRR_2016_ = 0.91 (0.91 to 0.91). Medication use decreased, IRR_2016_ = 0.82 (0.82 to 0.82).

**Conclusions:**

Our measure of post-discharge mortality was halved during the study period indicating improved health. Overall health service utilisation did not change much, but the type of utilisation changed, and the development over time differed between subgroups defined by age and chronic disease status. Our findings call for considerations about the benefit of increased specialisation and increased use of health services among ‘healthy’ children not suffering from chronic disease.

## Introduction

In Denmark the under-five mortality rate is approaching the absolute zero [1]. Since decreasing mortality reflects improved health, this is a success. At the same time, total expenses to health services increase [2]. On one hand it could be argued that mortality decreased because of increased supply and utilisation of health services. On the other hand, it could be argued that extensive utilisation of health services is no longer necessary. Not all physician contacts of under-fives concern potentially life-threatening conditions, but parents may allow little room for uncertainty, and the judgement of a physician might be sought.

To prioritise and organise our public health services in a way that gives the population the best and most efficient treatment and care and at the same time allocates the limited resources sensibly and evidence-based, it is helpful to first establish knowledge of the actual use of child health services in Denmark. Thus, the aim of this descriptive register-based study was to identify the level of health service utilisation in Danish children below 5 years of age born 1994–2016 and compare this with the development over the years of under-five mortality in the same population.

### Background information on Danish health services

The present study of health service utilisation among Danish children 1999–2016 is based on information from two main sources: general practice and the hospital system. Nearly all Danish residents (98%) are listed with a general practitioner (GP). The GP has the role of a family doctor and does the routine health checks and vaccination of the children on the list. GPs are freely and directly accessible and delivers primary medical care, and serves as a gatekeeper to specialists, e.g. paediatricians and orthopaedic surgeons, and hospital services [3]. ENT-specialists and ophthalmologists are available to patients without referral from the GP. Child health services are financed through taxes the exceptions being patient co-payment of prescription medicines and. The out-of-hours (OOH) service is organised in bigger schemes in the Danish regions and run by the GPs except in The Capital Region where a new Medical emergency Helpline system was introduced in 2014Text A in S1 File. Beside the change in the OOH service the primary care system has been stable in study period. The public hospital system is also free of charge after referral from a GP or another specialist. During the study period the largest structural change in the Danish health system was the reform of 2007 resulting in a merge of counties into the five larger administrative regions responsible for primary as well as secondary care, i.e. office medical specialists and hospitals which in addition underwent increased specialisation [4].

### Prior studies

Studies in other high-income settings indicated that the health service utilisation among Danish children is relatively high. For instance a study found a relatively low incidence of contacts general practitioners among Norwegian children [5]. Similarly, various Swedish studies indicated relatively low rates of different services such as hospitalisations for infection, outpatient contacts and antibiotics prescriptions compared to the level among Danish children [6–8]. On the other hand a study comparing use of antibiotic prescriptions among Swedish and Estonian children suggested that the Danish level was between that of these two countries respectively [9]. However, studies on all types of prescribed medications are sparse.

### The aims of the present study

The present study aimed at describing health service utilisation among Danish children 0 to 5 years of age from 1999 to 2016 using all register-based data available. We presented all hospital contacts and contacts with general practitioners and specialists both separately and as one composite measure. The latter provided a summary of total contacts with the health system over time and allowed comparison with the mortality trend. Further, the rates of prescribed, dispensed medications (all ATC-types) were presented.

Mortality is primarily presented as *post-discharge* mortality defined as mortality taking place after the first discharge from the hospital to the home after birth. This excludes children with life-threatening illness from birth. The secular trend of the conventional post-neonatal mortality rate is affected by two factors: first, changes in pre- and perinatal management of foetuses with serious malformations and born prematurely at the margin of viability, and second, the fact that deaths in premature children and children with malformations are increasingly often delayed to after 28 days, the conventional limit of the neonatal period. Hence in this report, we focus on mortality in infants who were discharged to the home at some time after birth.

## Methods

### Register data

The data were obtained from the Danish health registers as well as the register on deaths from Statistics Denmark [10] (Table 1). Individual level data were obtained using the Central Person Registration number (CPR) [11]. The health registers included The National Patient Register [12], The National Prescription Registry [13], and The Danish National Health Service Register [14].

**Table 1:** Health registers used in the study.

Register	Outcome	Short description
Deaths	Mortality	All deaths among Danish residents.
The National Patient Register	Inpatient and outpatient contacts	National register of activity within the Danish hospitals
The Register of Medicinal Product Statistics	Dispensed prescribed medication use	Register of all purchases of human (and veterinary) drugs. Does not contain individual level data on medication administered in-hospital
The Danish National Health Service Register	General practitioner, specialist and out-of-hours contacts	Register of all services which are reimbursed by the public health insurance.

#### Data quality

Reviews have concluded that the national Danish registers provide complete information and are valuable tools for epidemiological research [12,15,16]. Using the unique personal identification number of all Danish citizens the registers enable individual-level linkage between various sources of information including contacts with the health system. However, care need to be taken to conduct proper studies. The researcher needs to be aware of duplicate information as well as data consistency across time. The latter can be accommodated by thorough examination of the documentation on the authority websites [17,18]. This was also the case for this study which had to consider the introduction of neonatal hearing screenings which would otherwise affect the rate of outpatient contacts to an alarming extent. Another example of inconsistencies across time was provoked by the structural reform of the health system in 2007 which made it partially impossible to discern certain out-of-hours contacts from regular visits at the general practitioner. In addition, as mentioned in the Introduction, the new Medical emergency Helpline in The Capital Region of Denmark in 2014 introduced a new system of *acute outpatient contacts* which to a certain extent absorbed registration from the OOH service. Finally, the researcher must handle overlapping records for individuals regarding hospital admissions as well as migrations in and out of Denmark.

### Follow-up

The study included Danish children live-born in the period 1994–2016. Each child was followed from birth or time of first discharge until the first of the following events: death, five years of age, 31 December 2016, or migration. Migrations were handled by letting children enter the study at first immigration and leave and re-enter the study at emigrations and immigrations respectively. To ensure a comparable age composition across calendar years the results were presented from 1999 where the first children in the cohort reached five years of age.

### Definition of children with chronic disease

Children with chronic disease were identified using an existing algorithm based on registered diagnoses at discharge from hospital inpatient care [19]. The status was evaluated at first discharge to the home after birth, and subsequently updated each 1 January. Thus, it was possible that a given child belonged to the so-called ‘healthy’ group in one calendar year, but to the chronically ill group in the next, but once classified as chronically ill, the child remained in this group for the subsequent years. The group of children with chronic disease was defined to isolate the children not suffering from chronic disease to focus on the health care utilisation in this group, and the latter group will be referred to as the group of healthy children.

### Outcomes

Incidence rates of mortality, contacts with the health system, and prescribed medication were presented by calendar year. Contacts with the health system included inpatient and outpatient contacts, contacts with general practitioner (including out-of-hours), and specialist contacts. The composite measure of total contacts was also defined (please see below). Incidence rate ratios (IRR) with 1999 as the reference year were presented. The results are presented within subgroups of two different variables: age and chronic disease status. Results on differences between the genders are presented in the Supporting information (B-K Tables in S1 File).

#### Mortality

As mentioned in the introduction above the term *post-discharge mortality* was introduced and defined as the mortality among children who survived the first discharge from the hospital to the home after birth. As already mentioned, this approach excluded children dying from life-threatening illness present at birth.

#### Total contacts with the health system

The composite measure of total contacts with the health system is presented. Dispensed prescribed medications were not considered in this context. Viewing the temporal trend in this way was an attempt to disclose whether changes within the individual contact types (e.g. visits at general practitioner or specialist doctors) also resulted in a changed overall health service utilisation pattern. There was a possibility that the different types of contacts merely replaced each other over time whilst the demand for services remained constant.

#### Inpatient and outpatient contacts

Two different types of contacts from The National Patient Register were considered: inpatient (excluding administrative and birth-related ICD10-diagnoses) and outpatient contacts.

The term *post-discharge* was also applied for the inpatient contacts since a child does not contribute to the person time at risk before first discharge from the hospital to the home after birth.

During an inpatient hospitalisation the child was not at risk of experiencing another inpatient contact, but the same child could experience concurrent outpatient contacts.

If a child experienced a new inpatient contact less than one day after having been discharged from the latest hospital admission, the events were collapsed and only counted as one contact.

#### General practitioner and medical specialist contacts

Two types of contacts from primary care in The Danish National Health Service Register were considered: general practitioner contacts (including out-of-hours) and specialist contacts.

#### Medication

Data from The Danish National Prescription Registry on dispensed prescribed medication (all ATC-types) were considered. When analysing prescribed medication each child was instantaneously at risk of experiencing a new event.

### Subgroup variables

Each outcome was presented in total and by subgroups according to two variables: *age* which is divided in the three categories 0–28 days (neonatal age group), 28–365 days (middle age group), 1–5 years (oldest age group), and *presence of chronic disease* which is a dichotomous (yes/no) variable.

### Presentation and statistics

The present paper contains figures illustrating the results. Detailed tables containing the numbers which the figures are based on are presented in the Supporting information (A-K Tables in S1 File). Results are presented as incidence rate ratios estimated by Poisson regression. Subscripts indicate the comparison made, e.g. IRR_2013_ is the rate in 2013 divided by the rate in the reference year 1999. IRRs presented between subgroups—e.g. IRR_chronic_—refers to the overall comparison of rates between children with chronic disease and healthy children. In parenthesis the 95% Poisson confidence intervals (CI) are given. When the number of events was large the CIs were narrow. Sometimes the CIs were so narrow that the point estimate and the limits did not differ on the second decimal.

Note that the rates in the neonatal age group were based on only few days of time at risk. This means that this subgroup contributed relatively little to the total rate, which is a weighted average of the rates in the subgroups. Thus, the total rate will be much closer to the rates of the older age groups even if, for example, the neonatal rate was substantially larger.

## Results

Results on differences between the genders are presented in the Supporting information (B-K Tables in S1 File). Mortality and morbidity rates were consistently higher among boys, and the two subgroups experienced similar temporal trends.

### Post-discharge mortality

**Fig 1:**
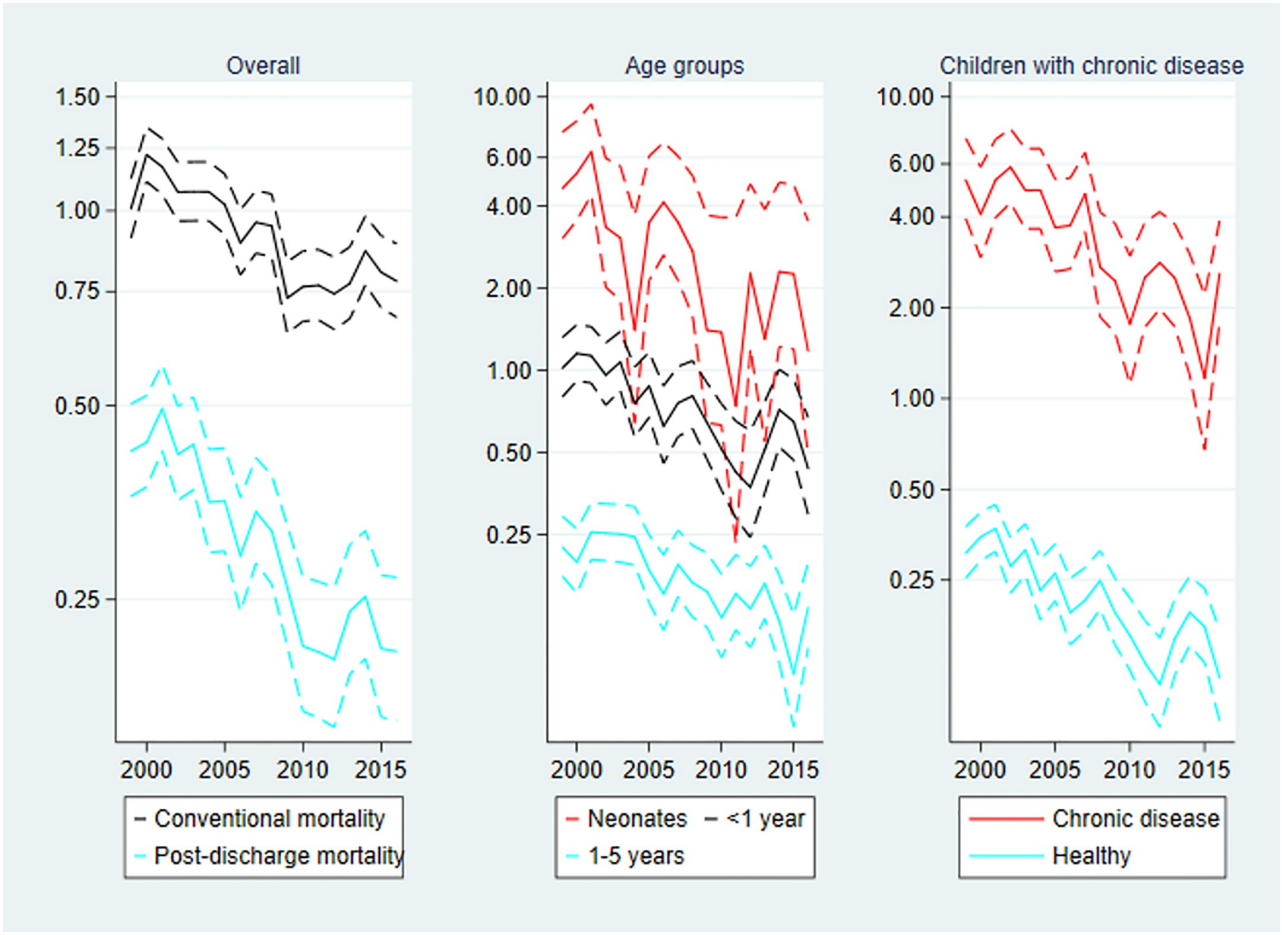
Post-discharge mortality per 1000 person-years, 1999–2016. Mortality rates with 95% confidence limits. Left panel: Overall post-discharge and conventional mortality. Centre panel: Post-discharge mortality by age group. Right panel: Post-discharge mortality by chronic disease status. Note the logarithmic scaling of the vertical axes and that the range of the leftmost vertical axis is different from those of the two other axes.

Mortality is presented in Fig 1 below. During the study period, the post-discharge mortality rate was halved from 0.42 deaths per 1000 person-years in 1999 to 0.21 deaths in 2016, IRR_2016_ = 0.49 (0.36–0.66).

In comparison conventional mortality was reduced by only 23 percent in the same period, IRR_2016_ = 0.77 (0.65–0.91). Detailed results on conventional mortality (and a comparison with the post-discharge approach) can be found in the Supporting information (B-D Tables in S1 File).

**Age**: The mortality rate was largest in the neonatal age group the rate being almost 4-fold that of the middle age group, and almost 16-fold that of the oldest age group. The trends over time differed between age groups, but the confidence intervals overlapped indicating no significant difference: IRR_2016_ was 0.25 (0.10–0.68) for neonates, 0.43 (0.27–0.69) for children aged 28–365 days, and 0.60 (0.39–0.93) for children aged 1–5 years.

**Chronic disease**: The mortality rate in the group of children with chronic disease was 15-fold that of the healthy children. The trends overt time differed between the children with vs. without chronic disease, but the confidence intervals overlapped: IRR_2016_ was 0.49 (0.30–0.79) for children with chronic disease, and 0.39 (0.26–0.57) for healthy children.

Care should be taken before interpreting the age-specific mortality curves (Fig 1). We observed that the mean age at first discharge to the home after birth decreased during the study period (data shown in the Supporting information, Table L in S1 File). This fact influenced the neonatal rates which seemed to increase dramatically. See the Discussion section for further elaboration on this aspect. Also note that the rates among the neonates were (by definition) based on relatively few person-years, which inflated the incidence rates.

### Total contacts with the health system

**Fig 2:**
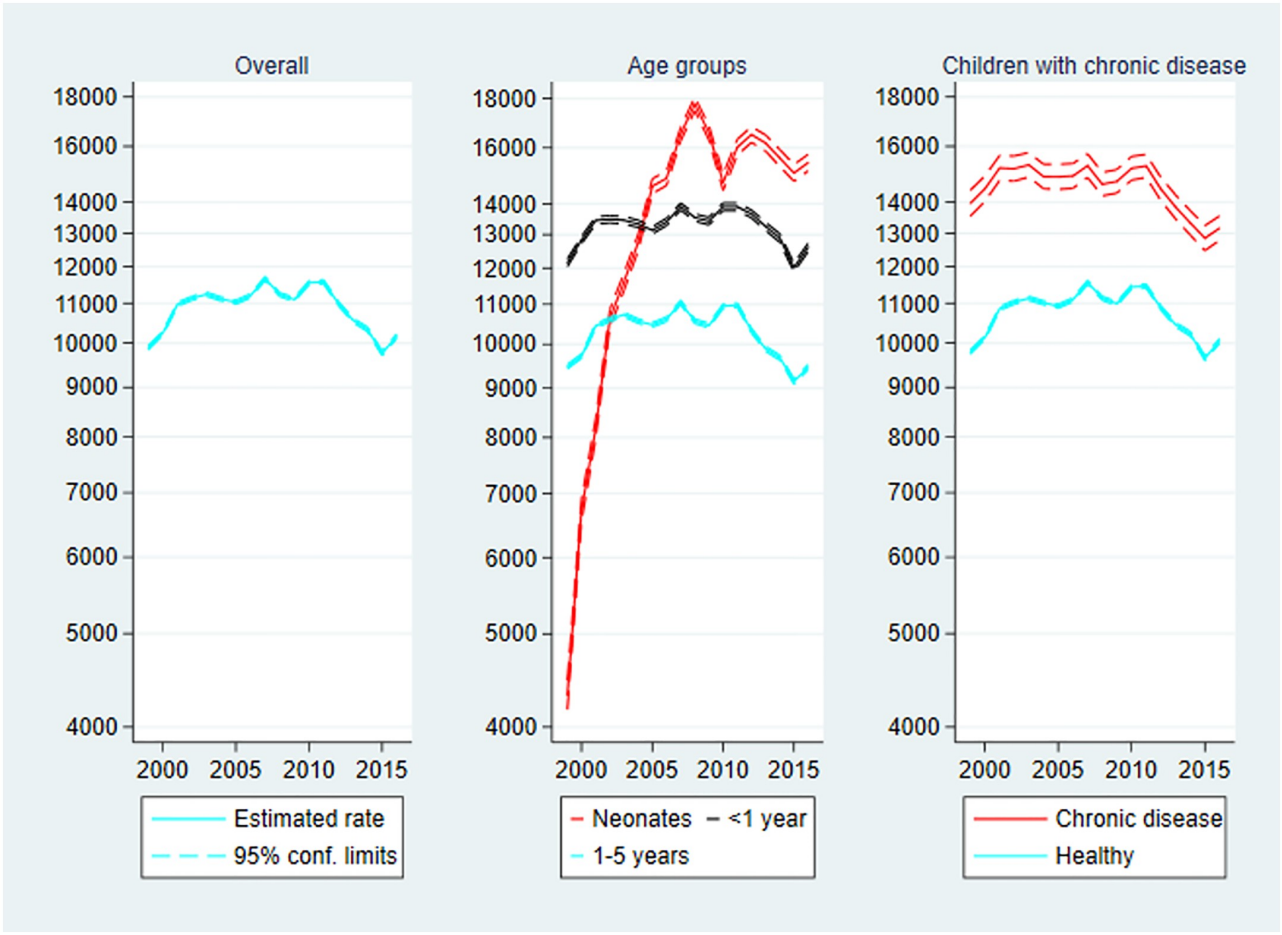
Total contacts per 1000 person-years, 1999–2016. Rate of total contacts with 95% confidence limits. Left panel: Overall total contacts. Centre panel: Total contacts by age group. Right panel: Total contacts by chronic disease status. Note the logarithmic scaling of the vertical axes.

Total contacts are presented in Fig 2 below. The rate of total contacts with the health system among Danish children aged 0–5 years at the end of the study period was very close to that of the beginning, IRR_2016_ = 1.02 (1.02–1.03). However, the rate was higher during the study period. The rate was highest in 2007 with 11,729 contacts per 1000 person-years, IRR_2007_ = 1.18 (1.18–1.18).

**Age**: The rate among neonates increased substantially during the initial of the study period from 4196 contacts per 1000 person-years in 1999 to 18,828 in 2007, IRR_2007_ = 4.49 (4.42–4.56). The increase compared to the reference year persisted through the study period, IRR_2016_ = 3.69 (3.63–3.75).

**Chronic disease**: The rate was consistently larger among children with chronic disease compared to healthy children, IRR_chronic_ = 1.34 (1.34–1.35). At the end of the study period the rate of contacts among children with chronic disease was lower than that of 1999, IRR_2016_ = 0.94 (0.93–0.94) meaning that the rate of total contacts increased slightly among healthy children, IRR_2016_ = 1.03 (1.03–1.03).

As for mortality the age-specific rates of total contacts (Fig 2) as well as the different types of contacts should be interpreted carefully. Recall that the mean age of discharge to the home after birth decreased during the study period (data shown in Supporting information, Table L in S1 File). In addition, the neonatal rates were based on fewer person-years. These two aspects inflated the neonatal rates.

### Inpatient contacts

**Fig 3:**
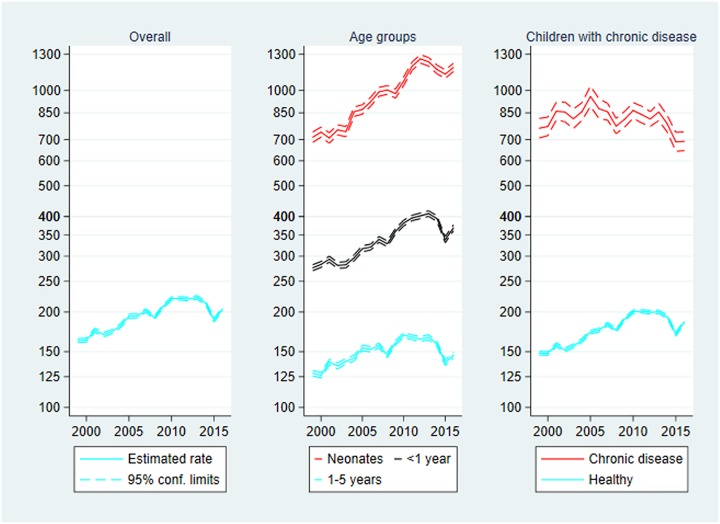
Inpatient contacts per 1000 person-years, 1999–2016. Rate of inpatient contacts with 95% confidence limits. Left panel: Overall inpatient contacts. Centre panel: Inpatient contacts by age group. Right panel: Inpatient contacts by chronic disease status. Note the logarithmic scaling of the vertical axes.

Inpatient contacts are presented in Fig 3 below. The rate of inpatient contacts increased during the study period from 162.68 contacts per 1000 person-years in 1999 to 204.58 in 2016, IRR_2016_ = 1.26 (1.24–1.27). The largest rate was observed in 2013 with 222.56 contacts per 1000 person-years, IRR_2013_ = 1.37 (1.35–1.38).

**Age**: The highest rate was found in the neonatal age group with 967.71 inpatient contacts per 1000 person-years. The rate was almost 3-fold that of the middle age group, and more than 6-fold that of the oldest age group. The increase over time also depended negatively on age, since IRR_2016_ = 1.66 (1.59–1.74) for neonates, IRR_2016_ = 1.34 (1.31–1.37) for the middle age group, and IRR_2016_ = 1.14 (1.12–1.16) for the oldest age group. That is the increase was most pronounced among neonates. Remember that these numbers represent only re-admission of children to hospital before 28 days of age, not initial admission to the maternity unit, nor admission to a paediatric or neonatal unit from the delivery unit or from the postnatal ward.

**Chronic disease**: The rate of inpatient contacts in children with chronic disease was 4-fold that of the otherwise healthy children, but the rate decreased over time among children with chronic disease, IRR_2016_ = 0.91 (0.88–0.94). Thus, the total increase over time mentioned above was driven by an increase among healthy children, IRR_2016_ = 1.26 (1.24–1.27).

### Outpatient contacts

**Fig 4:**
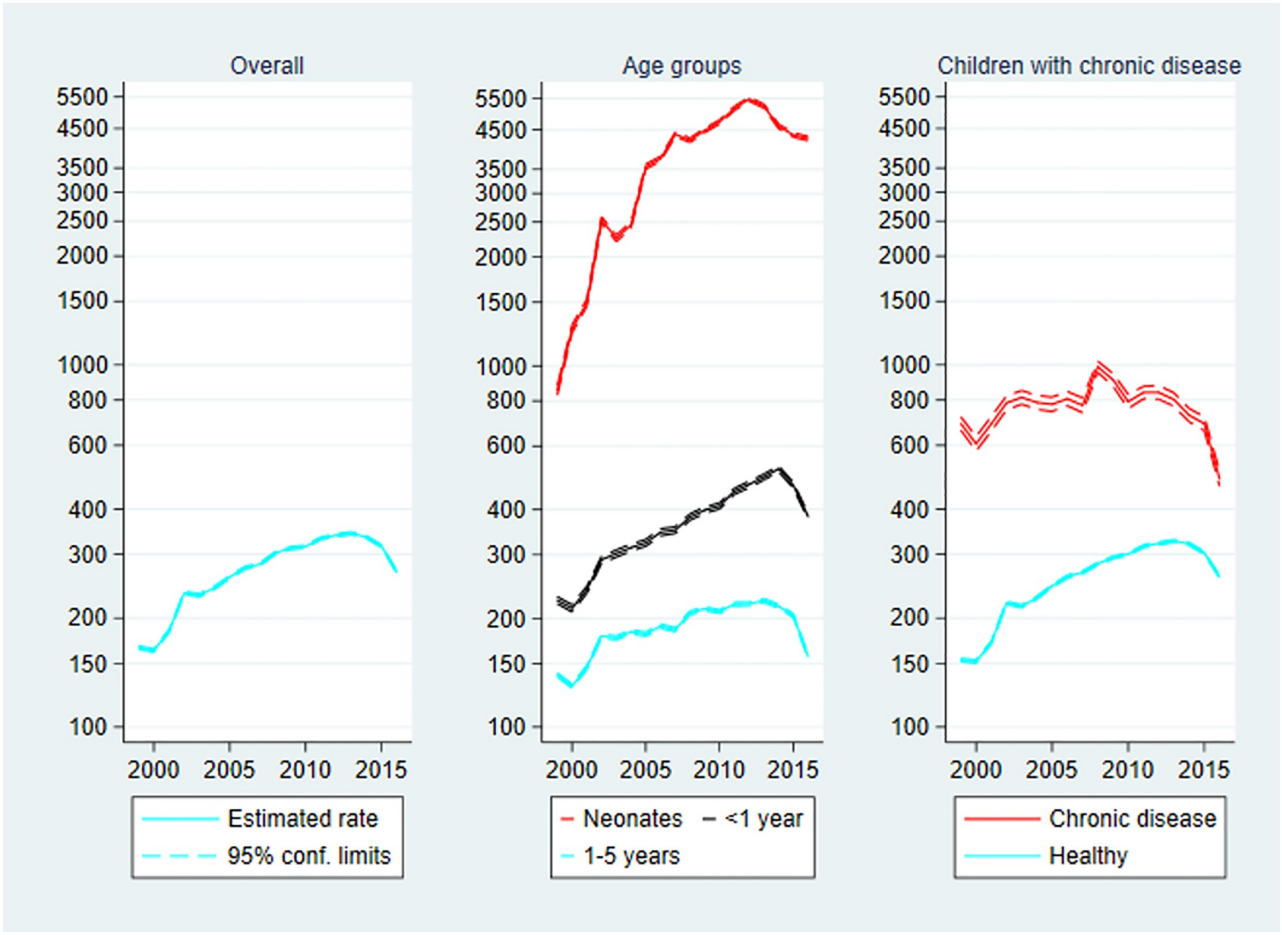
Outpatient contacts per 1000 person-years, 1999–2016. Rate of outpatient contacts with 95% confidence limits. Left panel: Overall outpatient contacts. Centre panel: Outpatient contacts by age group. Right panel: Outpatient contacts by chronic disease status. Note the logarithmic scaling of the vertical axes.

Outpatient contacts are presented in Fig 4 below. The rate of outpatient contacts increased during the study period from 165.79 contacts per 1000 person-years in 1999 to 268.08 in 2016, IRR_2016_ = 1.62 (1.60–1.63). The largest rate was observed in 2013 with 342.98 contacts per 1000 person-years, IRR_2013_ = 2.07 (2.05–2.09).

**Age**: As for inpatient contacts, the rate of outpatient contacts was largest in the neonatal age group: almost 10-fold that of the middle age group, and 19-fold that of the oldest age group. The increase in rates over time depended negatively on age, since IRR_2016_ = 4.98 (4.82–5.15) for neonates, IRR_2016_ = 1.71 (1.67–1.74) for the middle age group, and IRR_2016_ = 1.12 (1.11–1.14) for the oldest age group.

**Chronic disease**: The rate of outpatient contacts among children with chronic disease was 3-fold that of healthy children. However, the increase in rate over time was driven by an increase among healthy children, IRR_2016_ = 1.70 (1.68–1.72), whereas the rate of outpatient contacts decreased among children with chronic disease, IRR_2016_ = 0.70 (0.68–0.73).

### Contacts with general practitioner

**Fig 5:**
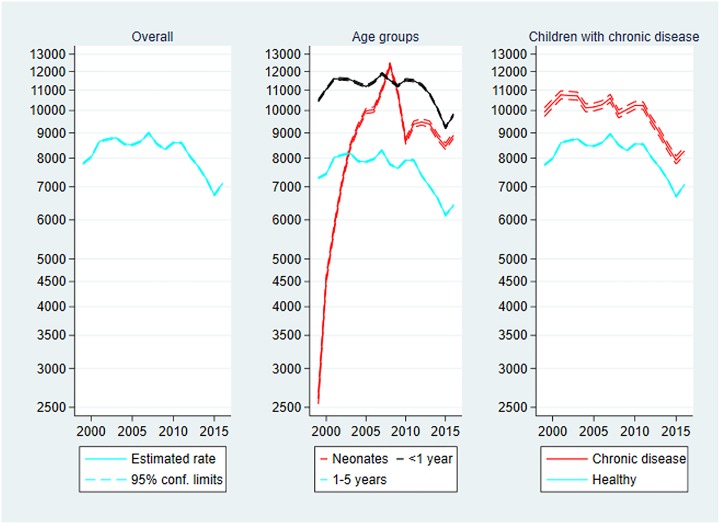
General practitioner contacts per 1000 person-years, 1999–2016. Rate of general practitioner contacts with 95% confidence limits. Left panel: Overall general practitioner contacts. Centre panel: General practitioner contacts by age group. Right panel: General practitioner contacts by chronic disease status. Note the logarithmic scaling of the vertical axes.

Contacts with general practitioner are presented in Fig 5 below. The rate of general practitioner contacts decreased from 8055.61 contacts per 1000 person-years in 1999 to 7116.44 in 2016, IRR_2016_ = 0.91 (0.91–0.91). The largest rate was found in 2007 with 9012.89 contacts per 1000 person-years, IRR_2007_ = 1.15 (1.15–1.16).

**Age**: The largest rate of general practitioner contacts was found in the middle age group (28–365 days of age) with 1108.49 per 1000 person-years, the IRRs compared to the other two age groups being IRR_neonates_ = 1.29 (1.29–1.29), and IRR_1–5 years_ = 1.46 (1.46–1.46) when compared to children aged 1–5 years.

Whereas the rates decreased over time within the two oldest age groups, as observed for inpatient and outpatient contacts, an increase was observed among neonates, IRR_2016_ = 3.41 (3.35–3.48).

**Chronic disease**: The rate was higher among children with chronic disease, IRR_chronic_ = 1.19 (1.19–1.20). However, the decrease over time was also more pronounced among children with chronic disease, IRR_2016_ = 0.83 (0.83–0.84) compared to IRR_2016_ = 0.91 (0.91–0.91) among healthy children.

### Contacts with medical specialists

**Fig 6:**
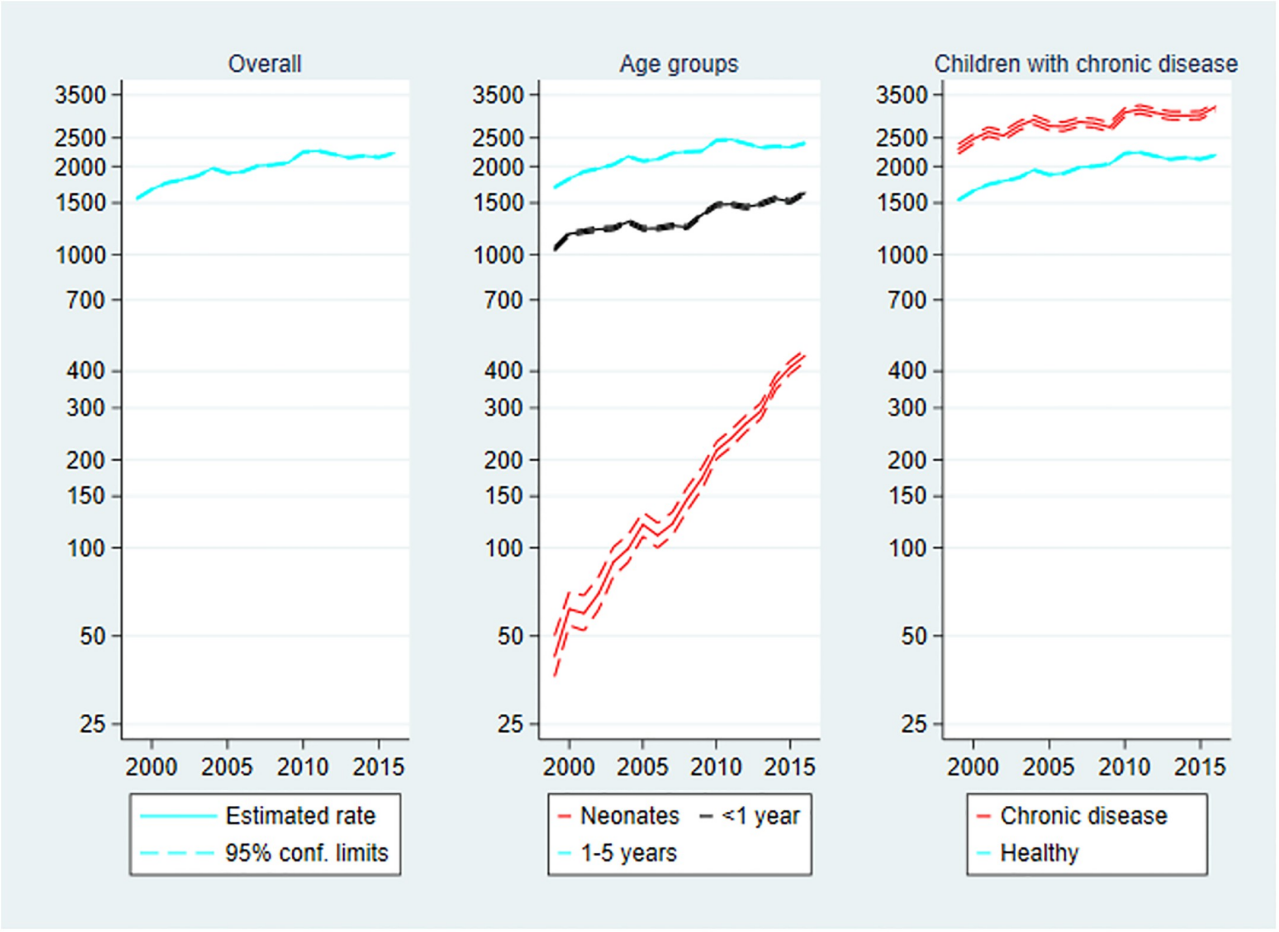
Specialist contacts per 1000 person-years, 1999–2016. Rate of medical specialist contacts with 95% confidence limits. Left panel: Overall medical specialist contacts. Centre panel: Medical specialist contacts by age group. Right panel: Medical specialist contacts by chronic disease status. Note the logarithmic scaling of the vertical axes.

Contacts with medical specialist are presented in Fig 6 below. The rate of contacts with specialists increased from 1554.34 contacts per 1000 person-years in 1999 to 2219.50 in 2016, IRR_2016_ = 1.43 (1.42–1.43).

**Age**: The rate of specialist contacts increased strongly with age. The rate in the oldest age group (1–5 years) was 2171.61 contacts per 1000 person-years, the IRRs compared to the other two age groups being IRR_28–365 days_ = 1.63 (1.63–1.64), and IRR_neonates_ = 12.10 (11.91–12.30).

However, the development over time differed substantially by age group. The increase was IRR_2016_ = 1.41 (1.41–1.41) in the oldest age group, and IRR_2016_ = 1.55 (1.53–1.57) in the group of children aged 28–365 days, whereas the increase in the neonatal age group was IRR_2016_ = 10.60 (9.21–12.19) which was consistent with the pattern of the other outcomes.

**Chronic disease**: The rate of specialist contacts was 45 percent higher among children with chronic disease than among healthy children, IRR_chronic_ = 1.45 (1.44–1.45). During the study period the increase in rate was similar between the two groups.

### Medication use

**Fig 7:**
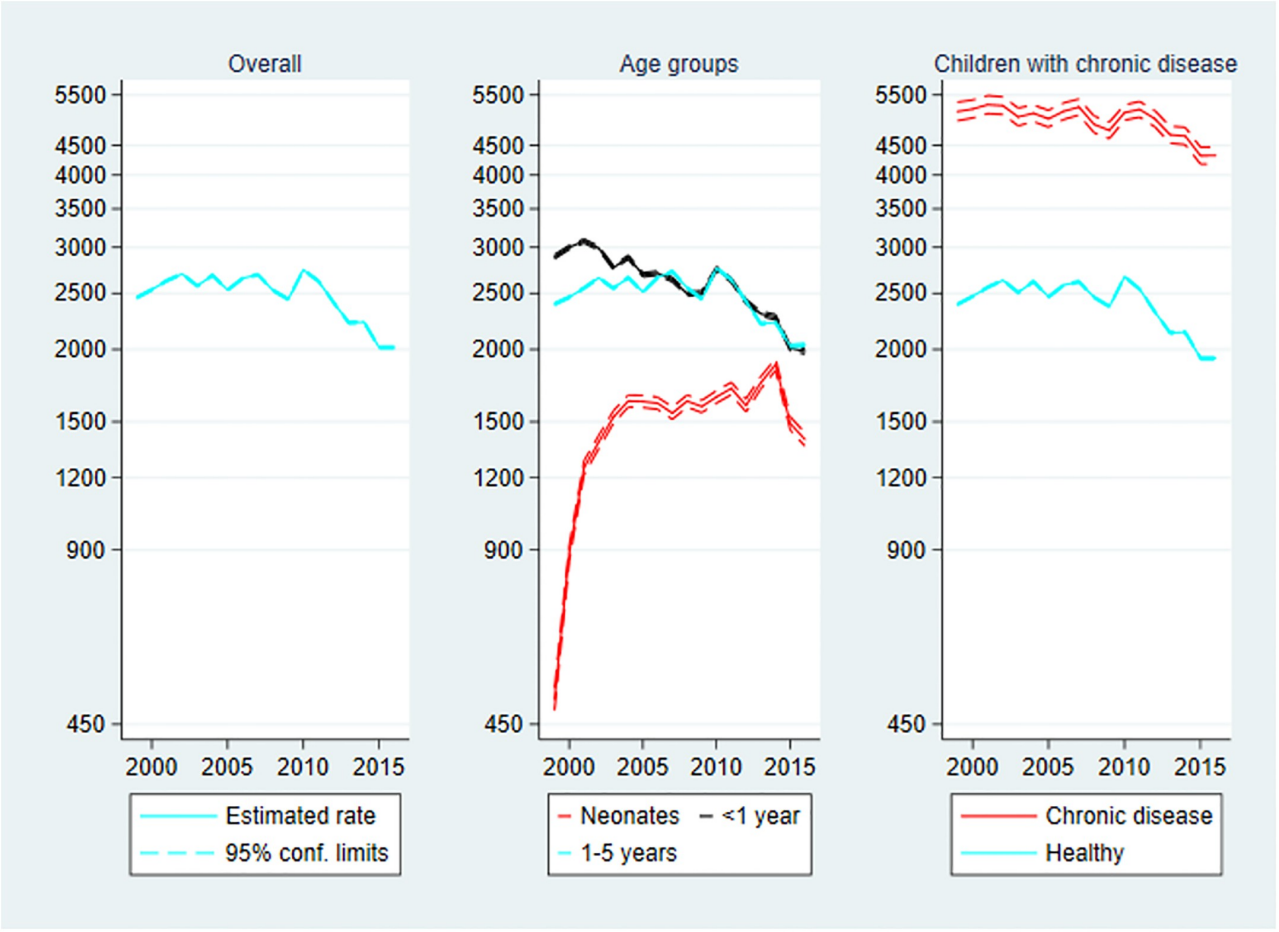
Prescriptions per 1000 person-years, 1999–2016. Rate of dispensed prescriptions with 95% confidence limits. Left panel: Overall medication use. Centre panel: Medication use by age group. Right panel: Medication use by chronic disease status. Note the logarithmic scaling of the vertical axes.

Illustrations on medication use are presented in Fig 7 below. The medication use decreased from 2457.75 prescriptions per 1000 person-years in 1999 to 2016.49 in 2016, IRR_2016_ = 0.82 (0.82–0.82). However, the rate increased in the beginning of the study period. The largest rate was found in 2010 with 2744.93 prescriptions per 1000 person-years, IRR_2010_ = 1.12 (1.11–1.12). Thus, the medication use decreased by almost 27 percent from 2010 to 2016.

**Age**: The largest use of prescribed medication was found in the group of children aged 28–365 days with 2677.05 prescriptions per 1000 person-years.

The medication use increased among neonates with IRR_2016_ = 2.81 (2.69–2.94) which was consistent with the findings of the contact types. In the two oldest age groups the medication use decreased: IRR_2016_ = 0.69 (0.68–0.69) in the middle age group, and IRR_2016_ = 0.85 (0.85–0.85) in the oldest age group.

**Chronic disease**: The medication use among children with chronic disease was approximately twice as large as that of healthy children, IRR_chronic_ = 2.05 (2.04–2.05). The development over time was similar in the two groups.

## Discussion

The objective of the present descriptive study was to obtain a quantification of post-discharge mortality and health service utilisation among Danish children aged 0–5 years over the last decades to assess whether the limited resources of the health system were spent efficiently. The primary findings were halved mortality, stable rate of total contacts (but more hospital admissions among healthy children), decreased medication use, increased health service utilisation among neonates, and increased specialisation in primary care.

### Post-discharge mortality

It is well-known that under-five mortality decreased in recent years [20]. This study shows that the development was even more positive when focusing on post-discharge mortality i.e. mortality among children who survived to be discharged to the home. The post-discharge mortality rate was halved during the study period 1999 to 2016.

This means that an increased proportion of infant mortality takes place at the hospital [1]. The distinction between pre- and post-discharge mortality is also made more relevant by the fact children with life-threatening illness at birth often survive the neonatal age, and thus the conventional neonatal and post-neonatal mortality rates are no longer suited to distinguish between the mortalities related to perinatal health on one side and infant health on the other. Our results suggest that these to sources of infant mortality and/or the health services addressing them did develop differently over the last 20 years.

The post-discharge mortality measure, however, is disturbed by the fact that some, albeit few, children are discharged home after birth for palliative care. Thus, one could argue that the *true* post-discharge mortality may be even lower.

### Contacts with the health system

The final level of the composite measure of total contacts with the health system was almost the same as in the beginning of the study period 1999. The initial increase was followed by a similar decrease. This raises the question if the demand of health services in the child population was stable whilst politicians and health professionals chose to refer children to different health services over time. The rate of inpatient and outpatient contacts increased, and the increase was driven by healthy children not suffering from chronic disease. The rate of contacts with general practitioner decreased, whereas contacts with specialists increased. This suggested a tendency to towards increased specialisation. In addition, the neonatal rates generally increased sharply.

### Medication use

During the study period the overall rate of dispensed prescribed medication decreased. Healthy children and children with chronic disease experienced a similar pattern over time. However, more and more prescriptions were issued to neonates. When mortality declines at the same time as medication use decreases one might consider the actual benefit of all the medication we prescribe to children in Denmark.

### Interpretation of results

First, post-discharge mortality was halved which indicates improved child health. The rate of total contacts remained stable and medication use decreased. The rate of contacts with general practitioner decreased whereas the contacts with specialists increased. This suggested a substitution of health services and increasing specialisation. However, from 2014 to 2016 the decrease in contacts within general practice had an obvious explanation through the introduction of a Medical emergency Helpline in The Capital Region of Denmark [21] representing more than 30% of the person-years in the study. A sub-analysis among the children outside the Capital Region found that the rate of general practitioner contacts did not follow the same pattern during the years in question.

For all outcomes the study found different developments by age (neonates) and chronic disease status. The general increase in inpatient contacts was not found among children with chronic disease. Thus, the increase was driven by the healthy children. The fact that otherwise healthy children were increasingly often admitted to hospital may indicate an element of so-called overdiagnosis or overtreatment [22].

The possible overdiagnosis of healthy children and the development towards increased specialisation are costly and thus the findings are important. Given that the minority of children with chronic disease experience fewer inpatient and outpatient contacts it is relevant to discuss whether the health system resources are allocated appropriately.

One could also hypothesise that development towards increased specialisation is partly a result of the health seeking behaviour of parents who demand the best possible treatment of their children.

The overall decreased use of dispensed prescribed medication—which is well-known to be driven by a decrease in antibiotic prescriptions [23]—suggested that the health of Danish children is not deteriorating. This adds to the discussion of why especially neonates were more frequently admitted to hospital and needed more medicine, and why healthy children instead of visiting a general practitioner attended specialists or were referred to outpatient clinics.

The increased utilisation of health services in neonates can partially be attributed to the fact that children were discharged from the hospital after birth increasingly early. In the earliest part of the study period the children were admitted to the hospital for a longer time and the incident cases would never be observed. If a child was discharged from hospital shortly after birth an outpatient or general practitioner contact could be one of the first events that the child would experience after discharge. In addition, more and more prescriptions are issued to neonates. It is of course possible that the health of neonates simply deteriorated during the study period. However alternative explanations appear more realistic. The sharp increase in health service utilisation among neonates took place before the concept of outpatient births were introduced in the Danish administrative regions in 2009 and 2010 which means that the increase is not explained by consequences of this initiative e.g. the fact that mother and child are discharged before breastfeeding is established [24].

Note that the decrease in total health service contacts observed from 2010 to 2016 coincided with a stagnation in the mortality rate which was otherwise decreasing sharply since 1999. The correspondence between mortality and development of health service utilisation is not one-to-one. Children are seen by health professionals even when the cause of the contact is not related to something potentially life-threatening.

### Prior studies

Various Danish studies include child populations and examine outcomes related to health service utilisation. The typical objective of these studies was to quantify one or more contrasts between subpopulations. Relevant outcomes for this purpose were contacts with the health system (e.g. contacts with general practitioner) or dispensed prescriptions of antibiotics. One study examined these outcomes among Danish children, and found an increased use in children with atopic disease [25]. A descriptive cross-sectional study of health service utilisation and medication use indicated a homogeneity across the five Danish administrative regions [26]. Another study illustrated how the incidence of contacts with general practitioner depends on age the pattern being a sharp increase in the first 1–2 years of living followed by an even sharper decrease [27].

Danish studies considering temporal trends have also been published. A study on hospitalisations due to infections among children aged 0–5 years from 1980 to 2001 pointed at an increased incidence which was largely driven by an increase in short term hospitalisations among the youngest children aged 0–1 year [28]. More recently a study on antibiotics prescriptions in Danish general practice 2004–13 found a stable high level of antibiotic use, but a decline in children aged 0-5-years [23].

Another study made a direct comparison of so-called out-of-hours contacts between Danish and Dutch children indicating a larger frequency among the Danes—especially in the 0-5-years age group [29].

### Strengths and limitations

The strengths of the present study include the population-based design, the long follow-up of 18 full calendar years, and the introduction of relevant measures of post-discharge mortality and total health service contacts.

The limitations include the fact that the data do not enable inference of the underlying mechanisms behind the findings that healthy children are more often admitted to hospital, and that contacts with the health system become increasingly specialised.

### Conclusion

The study found that post-discharge mortality among Danish children 0–5 years of age was halved from 1999 to 2016. The rate of total contacts with the health system remained stable, albeit with more healthy children admitted to hospital. However, fewer contacts for children with chronic disease, fewer contacts with general practitioners and more contacts with medical specialists suggested an increased element of specialisation among healthy children. At the same time prescribed medication use decreased. The quantification of the levels and developments enable discussions regarding the efficiency of the paediatric part of the Danish health care system.

The findings of the present study call for individual-level studies of determinants of health service utilisation to obtain an awareness of deliberate as well as latent factors or societal conditions affecting the health care utilisation over time. Also, studies considering if the observed patterns reflect sensible clinical practice are warranted.

## Ethics statement

The use of and access to all data were secured within the servers of Statistics Denmark. After linkage all data were fully pseudonymised. According to Danish law, ethics committee approval is not required for register-based studies.

## Supporting information

S1 File Supporting information. (DOCX)
