# Supplementary material for: Too much? Mortality and health service utilisation among Danish children 1999-2016: A register-based study
Source: PLoS One. 2019 Oct 30;14(10):e0224544. doi: 10.1371/journal.pone.0224544 (PMC6821095; doi:10.1371/journal.pone.0224544)
Supplement: S1 File — (DOCX) [file pone.0224544.s001.docx]

**Text A in S1 File. Supporting Information.**

This online supplement contains information related to the main article. Tables of results corresponding to the different outcomes are presented. The additions to the main article include results on conventional mortality (and a comparison with post-discharge mortality) and emergency contacts. Furthermore, the results are presented by gender as well. Clarifying remarks are attached to the outcomes when relevant.

After 2010 the rate of general practitioner contacts decreased sharply. After 2014 the decrease had an obvious explanation through the introduction of a Medical emergency Helpline in The Capital Region of Denmark (1). This aspect might also have influenced the attenuated outpatient contact rate which succeeded an otherwise sharp increase. It is also worth noticing that the five Danish regions with administrative responsibility of the health system were introduced in 2007 which possibly changed patterns of e.g. general practice (2).

**Table A in S1 File.** **Person-years among 0-5-year-old Danish children in the period 1999-2016.**

|  | **PY** | **Age** | | | **Gender** | | **Chronic disease** | |
| --- | --- | --- | --- | --- | --- | --- | --- | --- |
| **Year** | **Total** | **0-28 days** | **28-365 days** | **1-5 years** | **Male** | **Female** | **No** | **Yes** |
| 1999 | 338,453 | 5090 | 61,372 | 271,989 | 173,832 | 164,620 | 330,536 | 7915 |
| 2000 | 334,888 | 5134 | 61,247 | 268,506 | 172,043 | 162,845 | 326,796 | 8091 |
| 2001 | 333,499 | 5043 | 61,498 | 266,956 | 171,141 | 162,357 | 325,200 | 8298 |
| 2002 | 330,231 | 4925 | 59,758 | 265,547 | 169,322 | 160,908 | 321,850 | 8380 |
| 2003 | 327,930 | 4970 | 59,185 | 263,773 | 168,154 | 159,775 | 319,347 | 8581 |
| 2004 | 326,317 | 4955 | 59,881 | 261,479 | 167,319 | 158,997 | 317,744 | 8571 |
| 2005 | 324,683 | 4961 | 59,766 | 259,955 | 166,445 | 158,237 | 315,996 | 8686 |
| 2006 | 322,930 | 5010 | 59,744 | 258,175 | 165,427 | 157,503 | 314,124 | 8805 |
| 2007 | 322,991 | 4950 | 59,777 | 258,262 | 165,557 | 157,433 | 313,981 | 9009 |
| 2008 | 323,956 | 5013 | 60,108 | 258,833 | 165,945 | 158,010 | 314,775 | 9180 |
| 2009 | 323,534 | 4846 | 59,569 | 259,117 | 165,924 | 157,609 | 314,159 | 9373 |
| 2010 | 322,463 | 4907 | 58,735 | 258,820 | 165,452 | 157,010 | 312,818 | 9644 |
| 2011 | 319,497 | 4576 | 57,199 | 258,820 | 163,805 | 155,691 | 309,565 | 9931 |
| 2012 | 313,357 | 4468 | 54,104 | 254,784 | 160,615 | 152,741 | 303,076 | 10,280 |
| 2013 | 305,740 | 4317 | 53,170 | 248,251 | 156,729 | 149,010 | 295,383 | 10,356 |
| 2014 | 297,702 | 4382 | 51,992 | 241,326 | 152,464 | 145,237 | 287,430 | 10,270 |
| 2015 | 291,224 | 4446 | 52,703 | 234,073 | 149,209 | 142,014 | 280,944 | 10,279 |
| 2016 | 289,155 | 4698 | 55,171 | 229,284 | 148,418 | 140,737 | 278,761 | 10,393 |
| Total | 5,748,549 | 86,701 | 1,044,988 | 4,616,860 | 2,947,805 | 2,800,743 | 5,582,497 | 166,052 |

## Each cell contains the number of person-years (PY). Left-hand side of table: The total number of person-years is presented by calendar year. Right-hand side of table: For each calendar year the number of person-years is presented by age, gender, and presence of chronic disease.

**Text B in S1 File. Conventional mortality.**

From 1.01 deaths per 1000 person-years in 1999 the all-cause under-five mortality decreased to 0.78 in 2016, IRR_2016_ = 0.77 (0.65-0.91). The largest conventional mortality rate was observed in the calendar year 2000, IRR_2000_ = 1.21 (1.05-1.40), and the smallest was found in 2009 with IRR_2009_ = 0.73 (0.62-0.86). Based on the CIs the latter IRR is not discernible from the 2016-rate, though.

**Age:** The conventional mortality was dominated by the neonates. Comparisons with the group of neonates resulted in the ratios, IRR_28-365 days_ = 39.84 (36.27-43.75), and IRR_1-5 years_ = 222.97 (201.45-246.81). And the neonatal age group also experienced the smallest decrease in rate based on the point estimates of 2016, but the CIs overlap the IRR_2016_ being 0.83 (0.67-1.02) for neonates, 0.54 (0.36-0.81) for the middle age group, and 0.60 (0.39-0.93) for the oldest age group.

**Gender:** Overall boys had a 19 percent larger conventional mortality rate than girls, IRR_boys_ = 1.19 (1.10-1.28). In most calendar years, boys experienced the largest decrease in rate.

**Chronic disease:** The conventional mortality rate among children with chronic disease was more than 4-fold larger than that of healthy children. On the other hand, the children with chronic disease experienced the largest decrease in rate with e.g. IRR_2016_ = 0.55 (0.35-0.87) compared to IRR_2016_ = 0.70 (0.60-0.80) among healthy children but note that the confidence intervals overlapped.

Table B in S1 File. Conventional all-cause mortality from date of birth among 0-5-year-old Danish children 1999-2016.

|  | Events  PY  Rate (per 1000)  IRR (95% CI)  vs. 1999 | **Age** | | | **Gender** | | **Chronic disease** | |
| --- | --- | --- | --- | --- | --- | --- | --- | --- |
| **Year** | **Total** | **0-28 days** | **28-365 days** | **1-5 years** | **Male** | **Female** | **No** | **Yes** |
| **1999** | 341  1.01  Ref. year for IRR | 208  40.86 | 72  1.17 | 61  0.22 | 203  1.17 | 138  0.84 | 298  0.90 | 43  5.43 |
| **2000** | 409  1.22  1.21 (1.05-1.40) | 267  52.01  1.27 (1.06-1.53) | 88  1.44  1.22 (0.90-1.67) | 54  0.20  0.90 (0.62-1.29) | 236  1.37  1.17 (0.97-1.42) | 173  1.06  1.27 (1.01-1.58) | 374  1.14  1.27 (1.09-1.48) | 35  4.33  0.80 (0.51-1.24) |
| **2001** | 390  1.17  1.16 (1.00-1.34) | 228  45.21  1.11 (.92-1.34) | 93  1.51  1.29 (.95-1.75) | 69  0.26  1.15 (0.82-1.63) | 199  1.16  1.00 (0.82-1.21) | 191  1.18  1.40 (1.13-1.75) | 341  1.05  1.16 (1.00-1.36) | 49  5.90  1.09 (0.72-1.64) |
| **2002** | 353  1.07  1.06 (.91-1.23) | 218  44.26  1.08 (.90-1.31) | 68  1.14  0.97 (0.70-1.35) | 67  0.25  1.13 (0.80-1.59) | 190  1.12  0.96 (0.79-1.17) | 163  1.01  1.21 (.96-1.52) | 304  0.94  1.05 (0.89-1.23) | 49  5.85  1.08 (0.71-1.62) |
| **2003** | 351  1.07  1.06 (.92-1.23) | 202  40.64  0.99 (0.82-1.21) | 83  1.40  1.20 (0.87-1.64) | 66  0.25  1.12 (0.79-1.58) | 206  1.23  1.05 (0.86-1.27) | 145  0.91  1.08 (0.86-1.37) | 308  0.96  1.07 (0.91-1.25) | 43  5.01  0.92 (0.60-1.41) |
| **2004** | 349  1.07  1.06 (.91-1.23) | 223  45.00  1.10 (0.91-1.33) | 62  1.04  0.88 (0.63-1.24) | 64  0.25  1.09 (0.77-1.55) | 183  1.09  0.94 (0.77-1.14) | 166  1.04  1.24 (0.99-1.56) | 301  0.95  1.05 (0.90-1.23) | 48  5.60  1.03 (0.68-1.56) |
| **2005** | 332  1.02  1.01 (.87-1.18) | 219  44.14  1.08 (0.89-1.31) | 65  1.09  0.93 (0.66-1.30) | 48  0.19  0.82 (0.56-1.20) | 197  1.18  1.01 (0.83-1.23) | 135  0.85  1.02 (0.80-1.29) | 297  0.94  1.04 (0.89-1.22) | 35  4.03  0.74 (0.47-1.16) |
| **2006** | 288  0.89  0.89 (0.76-1.04) | 201  40.12  0.98 (0.81-1.19) | 48  0.80  0.68 (0.48-0.99) | 39  0.15  0.67 (0.45-1.01) | 163  0.99  0.84 (0.69-1.04) | 125  0.79  0.95 (0.74-1.21) | 254  0.81  0.90 (0.76-1.06) | 34  3.86  0.71 (0.45-1.11) |
| **2007** | 310  0.96  0.95 (0.82-1.11) | 195  39.39  0.96 (0.79-1.17) | 64  1.07  0.91 (0.65-1.28) | 51  0.20  0.88 (0.61-1.28) | 179  1.08  0.93 (0.76-1.13) | 131  0.83  0.99 (0.78-1.26) | 265  0.84  0.94 (0.79-1.10) | 45  4.99  0.92 (0.61-1.40) |
| **2008** | 307  0.95  0.94 (0.81-1.10) | 203  40.49  0.99 (0.82-1.20) | 61  1.02  0.87 (0.62-1.22) | 43  0.17  0.74 (0.50-1.09) | 179  1.08  0.92 (0.76-1.13) | 128  0.81  0.97 (0.76-1.23) | 276  0.88  0.97 (0.83-1.14) | 31  3.38  0.62 (0.39-0.99) |
| **2009** | 237  0.73  0.73 (0.62-0.86) | 147  30.33  0.74 (0.60-0.92) | 49  0.82  0.70 (0.49-1.01) | 41  0.16  0.71 (0.47-1.05) | 119  0.72  0.61 (0.49-0.77) | 118  0.75  0.89 (0.70-1.14) | 209  0.67  0.74 (0.62-.88) | 28  2.99  0.55 (0.34-0.89) |
| **2010** | 246  0.76  0.76 (0.64-0.89) | 161  32.80  0.80 (0.65-0.99) | 53  0.90  0.77 (0.54-1.10) | 32  0.12  0.55 (0.36-0.85) | 138  0.83  0.71 (0.58-0.89) | 108  0.69  0.82 (0.64-1.05) | 223  0.71  0.79 (0.66-0.94) | 23  2.38  0.44 (0.26-0.73) |
| **2011** | 245  0.77  0.76 (0.65-0.90) | 165  36.05  0.88 (0.72-1.08) | 41  0.72  0.61 (0.42-0.90) | 39  0.15  0.67 (0.45-1.01) | 129  0.79  0.67 (0.54-0.84) | 116  0.75  0.89 (0.69-1.14) | 215  0.69  0.77 (0.65-0.92) | 30  3.02  0.56 (0.35-0.89) |
| **2012** | 233  0.74  0.74 (0.62-0.87) | 167  37.38  0.91 (0.75-1.12) | 31  0.57  0.49 (0.32-0.74) | 35  0.14  0.61 (0.40-0.93) | 119  0.74  0.63 (0.51-0.80) | 114  0.75  0.89 (0.69-1.14) | 202  0.67  0.74 (0.62-0.88) | 31  3.02  0.56 (0.35-0.88) |
| **2013** | 236  0.77  0.77 (0.65-0.90) | 154  35.67  0.87 (0.71-1.08) | 41  0.77  0.66 (0.45-0.96) | 41  0.17  0.74 (0.50-1.09) | 122  0.78  0.67 (0.53-0.83) | 114  0.77  0.91 (0.71-1.17) | 209  0.71  0.78 (0.66-0.94) | 27  2.61  0.48 (0.30-0.78) |
| **2014** | 258  0.87  0.86 (0.73-1.01) | 176  40.16  0.98 (0.80-1.20) | 53  1.02  0.87 (0.61-1.24) | 29  0.12  0.54 (0.34-0.83) | 148  0.97  0.83 (0.67-1.03) | 110  0.76  0.90 (0.70-1.16) | 238  0.83  0.92 (0.77-1.09) | 20  1.95  0.36 (0.21-0.61) |
| **2015** | 234  0.80  0.80 (0.68-0.94) | 174  39.13  0.96 (0.78-1.17) | 42  0.80  0.68 (0.46-0.99) | 18  0.08  0.34 (0.20-0.58) | 125  0.84  0.72 (0.57-0.90) | 109  0.77  0.92 (0.71-1.18) | 220  0.78  0.87 (0.73-1.03) | 14  1.36  0.25 (0.14-0.46) |
| **2016** | 225  0.78  0.77 (0.65-0.91) | 159  33.84  0.83 (0.67-1.02) | 35  0.63  0.54 (0.36-0.81) | 31  0.14  0.60 (0.39-0.93) | 134  0.90  0.77 (0.62-0.96) | 91  0.65  0.77 (0.59-1.01) | 194  0.70  0.77 (0.64-0.92) | 31  2.98  0.55 (0.35-0.87) |
| **Total** | 5344  0.93 | 3467  39.99 | 1049  1.00 | 828  0.18 | 2969  1.01 | 2375  0.85 | 4728  0.85 | 616  3.71 |

Each cell contains (from top to bottom) (1) the number of events, (2) the incidence rate (per 1000 person-years), and (3) the incidence rate ratios (IRR) with 95% Poisson confidence intervals (CI) compared to the reference year 1999. Left-hand side of table: The totals of (1)-(4) are presented by calendar year. Right-hand side of table: For each calendar year (1)-(3) are presented by age group, gender, and presence of chronic disease.

**Text C in S1 File. Post-discharge mortality.**

**Gender:** Boys had a 16 percent larger mortality rate than girls, IRR_boys_ = 1.16 (1.06-1.28). No clear difference in trends between the sexes was observed.

Table C in S1 File. Mortality from date of first discharge from the hospital to the home after birth among 0-5-year-old Danish children in the period 1999-2016.

|  | Events  Rate (per 1000)  IRR (95% CI)  vs. 1999 | **Age** | | | **Gender** | | **Chronic disease** | |
| --- | --- | --- | --- | --- | --- | --- | --- | --- |
| **Year** | **Total** | **0-28 days** | **28-365 days** | **1-5 years** | **Male** | **Female** | **No** | **Yes** |
| 1999 | 143  0.42  Ref. year for IRR | 20  4.63 | 62  1.02 | 61  0.22 | 80  0.46 | 63  0.38 | 101  0.31 | 42  5.31 |
| 2000 | 146  0.44  1.05 (0.82-1.30) | 23  5.27  1.14 (0.62-2.07) | 70  1.15  1.13 (0.80-1.59) | 53  0.20  0.88 (0.61-1.27) | 83  0.48  1.05 (0.78-1.42) | 63  0.39  1.01 (0.71-1.43) | 113  0.35  1.13 (0.87-1.48) | 33  4.08  0.77 (0.49-1.21) |
| 2001 | 164  0.49  1.16 (0.93-1.46) | 27  6.30  1.36 (0.76-2.43) | 69  1.13  1.11 (0.79-1.56) | 68  0.26  1.14 (0.80-1.60) | 80  0.47  1.02 (0.74-1.38) | 84  0.52  1.35 (0.97-1.87) | 120  0.37  1.21 (0.93-1.57) | 44  5.31  1.00 (0.65-1.53) |
| 2002 | 138  0.42  0.99 (0.78-1.25) | 14  3.34  0.72 (0.36-1.43) | 57  0.96  0.94 (0.66-1.35) | 67  0.25  1.12 (0.80-1.59) | 69  0.41  0.89 (0.64-1.22) | 69  0.43  1.12 (0.80-1.58) | 89  0.28  0.90 (0.68-1.20) | 49  5.85  1.10 (0.73-1.66) |
| 2003 | 142  0.43  1.02 (0.81-1.29) | 13  3.05  0.66 (0.33-1.32) | 63  1.07  1.05 (0.74-1.49) | 66  0.25  1.12 (0.80-1.59) | 83  0.50  1.07 (0.79-1.46) | 59  0.37  0.96 (0.68-1.38) | 100  0.31  1.02 (0.78-1.35) | 42  4.90  0.92 (0.61-1.42) |
| 2004 | 115  0.35  0.83 (0.65-1.07) | 6  1.40  0.30 (0.12-0.75) | 45  0.76  0.74 (0.51-1.09) | 64  0.25  1.09 (0.77-1.55) | 57  0.34  0.74 (0.53-1.04) | 58  0.37  0.95 (0.67-1.36) | 73  0.23  0.75 (0.56-1.02) | 42  4.91  0.92 (0.60-1.42) |
| 2005 | 115  0.36  0.84 (0.66-1.07) | 15  3.48  0.75 (0.38-1.47) | 52  0.87  0.86 (0.60-1.24) | 48  0.18  0.82 (0.56-1.20) | 73  0.44  0.95 (0.69-1.31) | 42  0.27  0.69 (0.47-1.02) | 83  0.26  0.86 (0.64-1.15) | 32  3.69  0.69 (0.44-1.10) |
| 2006 | 94  0.29  0.69 (0.53-0.89) | 18  4.12  0.89 (0.47-1.68) | 37  0.62  0.61 (0.41-0.92) | 39  0.15  0.67 (0.45-1.01) | 51  0.31  0.67 (0.47-0.95) | 43  0.27  0.71 (0.48-1.05) | 61  0.20  0.64 (0.46-0.87) | 33  3.75  0.71 (0.45-1.11) |
| 2007 | 110  0.34  0.81 (0.63-1.03) | 15  3.48  0.75 (0.38-1.47) | 45  0.76  0.75 (0.51-1.09) | 50  0.19  0.86 (0.59-1.25) | 71  0.43  0.93 (0.68-1.28) | 39  0.25  0.65 (0.43-0.96) | 67  0.21  0.70 (0.51-0.95) | 43  4.77  0.90 (0.59-1.38) |
| 2008 | 103  0.32  0.76 (0.58-0.97) | 12  2.73  0.59 (0.29-1.20) | 48  0.80  0.79 (0.54-1.15) | 43  0.17  0.74 (0.50-1.09) | 57  0.35  0.75 (0.53-1.05) | 46  0.29  0.76 (0.52-1.11) | 78  0.25  0.81 (0.60-1.09) | 25  2.73  0.51 (0.31-0.84) |
| 2009 | 84  0.26  0.61 (0.47-0.80) | 6  1.40  0.30 (0.12-0.75) | 38  0.64  0.63 (0.42-0.95) | 40  0.15  0.69 (0.46-1.03) | 39  0.24  0.51 (0.35-0.75) | 45  0.29  0.75 (0.52-1.09) | 61  0.20  0.64 (0.46-0.87) | 23  2.46  0.46 (0.28-0.77) |
| 2010 | 68  0.21  0.50 (0.37-0.67) | 6  1.38  0.30 (0.12-0.74) | 30  0.51  0.51 (0.33-0.78) | 32  0.12  0.55 (0.36-0.85) | 39  0.24  0.51 (0.35-0.75) | 29  0.19  0.48 (0.31-0.75) | 51  0.16  0.53 (0.38-0.75) | 17  1.76  0.33 (0.19-0.58) |
| 2011 | 66  0.21  0.49 (0.37-0.65) | 3  0.74  0.16 (0.05-0.54) | 24  0.42  0.42 (0.26-0.67) | 39  0.15  0.67 (0.45-1.01) | 37  0.23  0.49 (0.33-0.72) | 29  0.19  0.49 (0.31-0.76) | 41  0.13  0.43 (0.30-.062) | 25  2.52  0.47 (0.29-0.78) |
| 2012 | 63  0.20  0.48 (0.35-0.64) | 9  2.26  0.49 (0.22-1.07) | 20  0.37  0.37 (0.22-0.61) | 34  0.13  0.60 (0.39-0.91) | 32  0.20  0.43 (0.29-0.65) | 31  0.20  0.53 (0.34-0.81) | 34  0.11  0.37 (0.25-.054) | 29  2.82  0.53 (0.33-0.85) |
| 2013 | 73  0.24  0.56 (0.43-0.75) | 5  1.30  0.28 (0.11-.075) | 27  0.51  0.50 (0.32-0.79) | 41  0.17  0.74 (0.50-1.09) | 39  0.25  0.54 (0.37-0.79) | 34  0.23  0.60 (0.39-0.90) | 47  0.16  0.52 (0.37-0.74) | 26  2.51  0.47 (0.29-0.77) |
| 2014 | 75  0.25  0.60 (0.45-0.79) | 9  2.29  0.50 (.23-.1.09) | 37  0.72  0.70 (0.47-1.06) | 29  0.12  0.54 (0.34-0.83) | 40  0.26  0.57 (0.39-0.83) | 35  0.24  0.63 (0.42-0.95) | 56  0.20  0.64 (0.46-0.88) | 19  1.85  0.35 (0.20-0.60) |
| 2015 | 61  0.21  0.49 (0.37-0.67) | 9  2.25  0.49 (0.22-1.07) | 34  0.65  0.64 (0.42-0.97) | 18  0.08  0.34 (0.20-0.58) | 32  0.22  0.47 (0.31-0.70) | 29  0.20  0.53 (0.34-0.83) | 49  0.17  0.57 (0.41-0.80) | 12  1.17  0.22 (0.12-0.42) |
| 2016 | 60  0.21  0.49 (0.36-0.66) | 5  1.18  0.25 (0.10-0.68) | 24  0.44  0.43 (0.27-0.69) | 31  0.14  0.60 (0.39-0.93) | 39  0.26  0.57 (0.39-0.83) | 21  0.15  0.39 (0.24-0.64) | 33  0.12  0.39 (0.26-0.57) | 27  2.60  0.49 (0.30-0.79) |
| Total | 1820  0.32 | 215  2.84 | 782  0.75 | 823  0.18 | 1001  0.34 | 819  0.29 | 1257  0.23 | 563  3.39 |

Each cell contains (from top to bottom) (1) the number of events, (2) the incidence rate (per 1000 person-years), and (3) the incidence rate ratios (IRR) with 95% Poisson confidence intervals (CI) compared to the reference year 1999. Left-hand side of table: The totals of (1)-(3) are presented by calendar year. Right-hand side of table: For each calendar year (1)-(3) are presented by age group, gender, and presence of chronic disease.

**Text D in S1 File. Comparison of conventional vs. post-discharge mortality**

In Table D in S1 File the two types of all-cause mortality, conventional and post-discharge, are compared between different subgroups of the considered variables.

Table D in S1 File. Mortality among 0-5-year-old Danish children in the period 1999-2016.

| **Variable** | **Group** | **Conventional mortality rate** | **Post-discharge mortality rate** |
| --- | --- | --- | --- |
|  |  |  |  |
| **Total** |  | 0.93 | 0.32 |
|  |  |  |  |
| **Age** | 0-28 days | 39.99 | 2.84 |
|  | 28-365 days | 1.00 | 0.75 |
|  | 1-5 years | 0.18 | 0.18 |
|  |  |  |  |
| **Gender** | Male | 1.01 | 0.34 |
|  | Female | 0.85 | 0.29 |
|  |  |  |  |
| **Chronic disease** | No | 0.85 | 0.23 |
|  | Yes | 3.71 | 3.39 |

For each of the two definitions of mortality the total mortality rate in the period 1999-2016 is presented by age, gender, and presence of chronic disease. Conventional mortality refers to mortality from date of birth. Post-discharge mortality refers to mortality from date of first discharge from the hospital to the home after birth.

**Text E in S1 File. Total contacts.**

Total contacts consist of the following types of contacts: inpatient, outpatient, emergency, general practitioner (including out-of-hours) and medical specialist.

**Gender:** The rate of total contacts was consistently larger among boys, IRR_boys_ = 1.12 (1.12-1.12). The development over time was similar in the two groups.

Table E in S1 File. Total contacts among 0-5-year-old Danish children in the period 1999-2016.

|  | Events  Rate (per 1000)  IRR (95% CI)  vs. 1999 | **Age** | | | **Gender** | | **Chronic disease** | |
| --- | --- | --- | --- | --- | --- | --- | --- | --- |
| **Year** | **Total** | **0-28 days** | **28-365 days** | **1-5 years** | **Male** | **Female** | **No** | **Yes** |
| 1999 | 3,314,992  9948.09  Ref. year for IRR | 21,263  4196.38 | 742,092  12,128.24 | 2,551,637  9437.13 | 1,793,191  10,391.09 | 1,521,801  9369.70 | 3,205,288  9817.34 | 109,704  14,036.06 |
| 2000 | 3,395,614  10,272.96  1.03 (1.03-1.03) | 33,681  7763.30  1.85 (1.82-1.88) | 781,482  12,855.93  1.06 (1.05-1.06) | 2,580,451  9720.24  1.03 (1.02-1.03) | 1,844,718  10,910.64  1.05 (1.05-1.05) | 1,550,896  9650.79  1.03 (1.03-1.04) | 3,279,513  10,210.03  1.04 (1.04-1.04) | 116,101  14,597.50  1.04 (1.03-1.04) |
| 2001 | 3,616,991  10,969.07  1.10 (1.10-10) | 40,407  8050.80  1.92 (1.89-1.95) | 822,263  13,449.41  1.11 (1.10-1.11) | 2,754,321  10,449.33  1.10 (1.10-1.10) | 1,964,254  11,609.16  1.12 (1.11-1.12) | 1,652,737  10,348.66  1.11 (1.11-1.11) | 3,492,182  10,921.52  1.12 (1.11-1.12) | 124,809  15,184.07  1.08 (1.07-1.09) |
| 2002 | 3,633,879  11,188.28  1.12 (1.12-1.13) | 51,542  12,366.18  2.95 (2.90-3.00) | 801,514  13,486.11  1.11 (1.11-1.11) | 2,780,823  10,640.04  1.12 (1.12-112) | 1,971,008  11,841.15  1.14 (1.14-1.14) | 1,662,871  10,453.73  1.12 (1.12-1.12) | 3,507,971  11,093.46  1.13 (1.13-1.14) | 125,908  15,310.23  1.09 (1.08-1.10) |
| 2003 | 3,641,261  11,239.97  1.13 (1.13-1.13) | 56,846  11,493.27  2.74 (2.70-2.78) | 791,905  13,475.48  1.11 (1.11-1.11) | 2,792,510  10,734.02  1.13 (1.13-1.13) | 1,975,575  11,951.99  1.15 (1.15-1.15) | 1,665,686  10,576.12  1.13 (1.13-1.14) | 3,511,215  11,157.20  1.14 (1.14-1.14) | 130,046  15,344.28  1.09 (1.08-1.10) |
| 2004 | 3,580,875  11,172.421.11 1.12 (1.12-1.12) | 62,288  12,633.93  3.01 (2.97-3.06) | 794,170  13,360.56  1.10 (1.10-1.10) | 2,724,417  10,569.44  1.12 (1.11-1.12) | 1,943,497  11,819.33  1.14 (1.13-1.14) | 1,637,378  10,469.23  1.12 (1.12-1.12) | 3,454,623  11,010.82  1.12 (1.12-1.13) | 126,252  14,877.69  1.06 (1.05-1.06) |
| 2005 | 3,534,308  11,079.89  1.11 (1.11-1.12) | 71,459  14,476.47  3.45 (3.40-3.50) | 780,045  13,134.57  1.08 (1.08-1.08) | 2,682,804  10,500.17  1.11 (1.11-1.11) | 1,914,383  11,686.25  1.12 (1.12-1.12) | 1,619,925  10,399.18  1.11 (1.11-1.12) | 3,406,485  10,923.57  1.12 (1.11-1.12) | 127,823  14,910.54  1.06 (1.05-1.07) |
| 2006 | 3,567,648  11,197.89  1.13 (1.12-1.13) | 73,463  14,750.21  3.52 (3.46-3.57) | 795,445  13,484.99  1.11 (1.11-1.11) | 2,698,740  10,614.78  1.12 (1.12-1.12) | 1,932,151  11,876.42  1.14 (1.14-1.14) | 1,635,497  10,555.99  1.13 (1.13-1.13) | 3,437,766  11,128.08  1.14 (1.14-1.14) | 129,882  14,899.79  1.06 (1.05-1.07) |
| 2007 | 3,715,992  11,729.42  1.18 (1.18-1.18) | 80,651  18,828.14  4.49 (4.42-4.56) | 825,580  13,905.18  1.14 (1.14-1.15) | 2,809,761  11,096.67  1.17 (1.17-1.17) | 2,004,794  12,281.35  1.18 (1.18-1.18) | 1,711,198  11,079.55  1.19 (1.18-1.19) | 3,579,938  11,635.50  1.19 (1.19-1.19) | 136,054  15,327.17  1.09 (1.08-1.10) |
| 2008 | 3,586,814  11,230.38  1.13 (1.13-1.13) | 87,969  17,656.09  4.21 (4.15-4.27) | 808,212  13,541.72  1.11 (1.11-1.12) | 2,690,633  10,597.18  1.12 (1.12-1.12) | 1,942,193  11,873.26  1.14 (1.14-1.14) | 1,644,621  10,586.69  1.13 (1.13-1.14) | 3,454,211  11,131.54  1.14 (1.14-1.14) | 132,603  14,641.46  1.04 (1.03-1.05) |
| 2009 | 3,541,174  11,162.67  1.12 (1.12-1.12) | 79,276  16,463.05  3.93 (3.87-3.98) | 794,065  13,424.20  1.10 (1.10-1.11) | 2,667,833  10,458.04  1.10 (1.10-1.11) | 1,923,500  11,818.64  1.14 (1.13-1.14) | 1,617,674  10,405.60  1.11 (1.11-1.12) | 3,404,690  11,021.86  1.13 (1.12-1.13) | 136,484  14,710.34  1.04 (1.04-1.05) |
| 2010 | 3,674,382  11,550.34  1.16 (1.16-1.16) | 71,262  14,606.32  3.48 (3.43-3.54) | 810,881  13,921.22  1.15 (1.14-1.15) | 2,792,239  10,984.43  1.16 (1.16-1.16) | 1,989,783  12,250.09  1.18 (1.17-1.18) | 1,684,599  10,873.91  1.17 (1.16-1.17) | 3,529,438  11,491.14  1.17 (1.17-1.18) | 144,944  15,200.85  1.08 (1.07-1.09) |
| 2011 | 3,646,454  11,605.97  1.17 (1.16-1.17) | 72,241  15,890.48  3.79 (3.73-3.85) | 789,146  13,968.76  1.15 (1.15-1.15) | 2,785,067  10,980.31  1.16 (1.16-1.16) | 1,969,499  12,199.10  1.17 (1.17-1.17) | 1,676,955  10,981.44  1.18 (1.17-1.18) | 3,496,471  11,487.43  1.17 (1.17-1.18) | 149,983  15,296.44  1.09 (1.08-1.09) |
| 2012 | 3,394,948  11,049.62  1.11 (1.11-1.11) | 72,733  18,410.71  4.39 (4.32-4.46) | 733,862  13,681.52  1.13 (1.12-1.13) | 2,588,353  10,330.85  1.09 (1.09-1.09) | 1,834,827  11,650.69  1.12 (1.12-1.12) | 1,560,121  10,396.10  1.11 (1.11-1.12) | 3,248,041  10,940.45  1.12 (1.12-1.12) | 146,907  14,485.08  1.03 (1.02-1.04) |
| 2013 | 3,182,564  10,596.47  1.07 (1.06-1.07) | 68,916  16,048.22  3.83 (3.77-3.89) | 700,224  13,344.78  1.10 (1.09-1.10) | 2,413,424  9890.41  1.04 (1.04-1.05) | 1,721,599  11,151.84  1.07 (1.07-1.07) | 1,460,965  10,004.14  1.07 (1.07-1.07) | 3,040,636  10,481.15  1.07 (1.07-1.07) | 141,928  13,872.8  0.98 (0.98-0.99) |
| 2014 | 3,031,077  10,392.99  1.04 (1.04-1.05) | 67,582  15,492.27  3.69 (3.64-3.75) | 666,491  12,891.62  1.06 (1.06-1.06) | 2,297,004  9710.26  1.02 (1.02-1.03) | 1,638,457  10,906.16  1.05 (1.05-1.05) | 1,392,620  9736.32  1.04 (1.04-1.05) | 2,895,562  10,284.85  1.05 (1.05-1.05) | 135,515  13,404.58  0.95 (0.94-0.96) |
| 2015 | 2,803,162  9761.25  0.98 (0.98-0.98) | 66,451  14,948.77  3.56 (3.51-3.62) | 630,575  12,094.06  1.00 (0.99-1.00) | 2,106,136  9146.99  0.97 (0.96-0.97) | 1,518,231  10,317.71  0.99 (0.99-0.99) | 1,284,931  9176.33  0.98 (0.98-0.99) | 2,672,416  9699.31  0.99 (0.99-0.99) | 130,746  12,847.62  0.91 (0.90-0.92) |
| 2016 | 2,825,802  10,191.84  1.02 (1.02-1.03) | 70,921  15,475.76  3.69 (3.63-3.75) | 675,560  12,288.84  1.01 (1.01-1.01) | 2,079,321  9483.78  1.00 (1.00-1.00) | 1,532,556  10,512.35  1.01 (1.01-1.01) | 1,293,246  9584.60  1.03 (1.02-1.03) | 2,693,417  10,079.22  1.03 (1.03-1.03) | 132,385  13,199.07  0.94 (0.93-0.94) |
| Total | 61,687,937  10,918.68 | 1,148,951  13,755.04 | 13,743,512  13,254.74 | 46,795,474  10,312.03 | 33,414,216  11,515.38 | 28,273,721  10,265.97 | 59,309,863  10,815.94 | 2,378,074  14,529.69 |

Each cell contains (from top to bottom) (1) the number of events, (2) the incidence rate (per 1000 person-years), and (3) the incidence rate ratios (IRR) with 95% Poisson confidence intervals (CI) compared to the reference year 1999. Left-hand side of table: The totals of (1)-(3) are presented by calendar year. Right-hand side of table: For each calendar year (1)-(3) are presented by age group, gender, and presence of chronic disease.

**Text F in S1 File. Inpatient contacts.**

From 2002 onwards, the registration of inpatient and outpatient contacts was changed. We defined the new contacts as outpatient contacts even though the procedure of registering these contacts as either inpatient or outpatient contacts differed between hospitals.

**Gender:** Overall the rate among boys was 30 percent larger than that among girls, IRR_2016_ = 1.30 (1.29-1.31). However, the increase was generally larger among girls with the IRR_2016_ being 1.32 (1.30-1.35) for girls and 1.21 (1.19-1.23) for boys.

Table F in S1 File. Inpatient contacts among 0-5-year-old Danish children in the period 1999-2016.

|  | Events  Rate (per 1000)  IRR (95% CI)  vs. 1999 | **Age** | | | **Gender** | | **Chronic disease** | |
| --- | --- | --- | --- | --- | --- | --- | --- | --- |
| **Year** | **Total** | **0-28 days** | **28-365 days** | **1-5 years** | **Male** | **Female** | **No** | **Yes** |
| 1999 | 54,153  162.68  Ref. year for IRR | 3057  712.42 | 16,730276.05 | 34,366  128.26 | 32,026  187.37 | 22,127  136.65 | 48,234  148.38 | 5919  760.21 |
| 2000 | 53,701  163.15  1.00 (0.99-1.01) | 3207  739.19  1.04 (0.99-1.09) | 17,096  282.66  1.02 (1.00-1.05) | 33,398  126.34  0.99 (0.97-1.00) | 31,586  186.82  1.00 (0.98-1.01) | 22,115  138.15  1.01 (0.99-1.03) | 47,576  148.12  1.00 (0.99-1.01) | 6125  770.10  1.01 (0.98-1.05) |
| 2001 | 57,399  175.23  1.08 (1.06-1.09) | 3017  708.18  0.99 (0.95-1.05) | 17,862  293.85  1.06 (1.04-1.09) | 36,520  139.11  1.08 (1.07-1.10) | 34,081  202.85  1.08 (1.07-1.10) | 23,318  146.15  1.07 (1.05-1.09) | 50,400  157.78  1.06 (1.05-1.08) | 6999  860.68  1.13 (1.09-1.17) |
| 2002 | 55,049  169.67  1.04 (1.03-1.06) | 3132  751.44  1.05 (1.00-1.11) | 16,599  280.81  1.02 (1.00-1.04) | 35,318  135.23  1.05 (1.04-1.07) | 32,447  195.17  1.04 (1.03-1.06) | 22,602  142.88  1.05 (1.03-1.07) | 48,012  151.83  1.02 (1.01-1.04) | 7037  855.69  1.13 (1.09-1.17) |
| 2003 | 55,892  173.49  1.07 (1.05-1.08) | 3140  741.66  1.04 (0.99-1.10) | 16,507  282.02  1.02 (1.00-1.04) | 36,245  139.73  1.09 (1.07-1.11) | 32,968  199.68  1.07 (1.05-1.08) | 22,924  145.96  1.07 (1.05-1.09) | 49,034  156.29  1.05 (1.04-1.07) | 6858  814.09  1.07 (1.03-1.11) |
| 2004 | 58,028  181.05  1.11 (1.10-1.13) | 3645  857.30  1.20 (1.15-1.26) | 17,569  296.67  1.07 (1.05-1.10) | 36,814  143.23  1.12 (1.10-1.13) | 33,669  204.98  1.09 (1.08-1.11) | 24,359  155.89  1.14 (1.12-1.16) | 50,809  162.79  1.10 (1.08-1.11) | 7219  857.85  1.13 (1.09-1.17) |
| 2005 | 61,811  193.97  1.19 (1.18-1.21) | 3737  871.70  1.22 (1.17-1.28) | 18,717  316.98  1.15 (1.12-1.17) | 39,357  154.13  1.20 (1.18-1.22) | 35,654  218.30  1.17 (1.15-1.18) | 26,157  168.38  1.23 (1.21-1.25) | 53,648  172.97  1.17 (1.15-1.18) | 8163  958.21  1.26 (1.22-1.30) |
| 2006 | 61,576  194.45  1.20 (1.18-1.21) | 3995  921.60  1.29 (1.23-1.36) | 18,915  320.66  1.16 (1.14-1.19) | 38,666  152.62  1.19 (1.17-1.21) | 35,607  219.56  1.17 (1.15-1.19) | 25,969  168.10  1.23 (1.21-1.25) | 54,011  175.35  1.18 (1.17-1.20) | 7565  875.64  1.15 (1.11-1.19) |
| 2007 | 63,970  202.12  1.24 (1.23-1.26) | 4242  990.30  1.39 (1.33-1.46) | 20,017  339.22  1.23 (1.20-1.25) | 39,711  156.83  1.22 (1.21-1.24) | 36,672  226.10  1.21 (1.19-1.23) | 27,298  176.91  1.29 (1.27-1.32) | 56,405  183.33  1.24 (1.22-1.25) | 7565  857.12  1.13 (1.09-1.17) |
| 2008 | 60,961  192.04  1.18 (1.17-1.19) | 4387  1004.31  1.41 (1.35-1.48) | 19,481  328.43  1.19 (1.17-1.21) | 37,093  146.18  1.14 (1.12-1.16) | 35,096  215.93  1.15 (1.14-1.17) | 25,865  166.97  1.22 (1.20-1.24) | 54,010  175.11  1.18 (1.17-1.19) | 6951  772.23  1.02 (0.98-1.05) |
| 2009 | 66,012  208.09  1.28 (1.26-1.29) | 4166  978.11  1.37 (1.31-1.44) | 21,142  359.67  1.30 (1.28-1.33) | 40,704  160.13  1.25 (1.23-1.27) | 38,342  235.81  1.26 (1.24-1.28) | 27,670  178.93  1.31 (1.29-1.33) | 58,541  190.05  1.28 (1.27-1.30) | 7471  811.53  1.07 (1.03-1.10) |
| 2010 | 69,783  220.55  1.36 (1.34-1.37) | 4643  1072.51  1.51 (1.44-1.58) | 22,148  382.02  1.38 (1.36-1.41) | 42,992  169.20  1.32 (1.30-1.34) | 39,911  245.94  1.31 (1.29-1.33) | 29,872  193.82  1.42 (1.39-1.44) | 61,577  200.63  1.35 (1.34-1.37) | 8206  865.32  1.14 (1.10-1.18) |
| 2011 | 69,247  221.03  1.36 (1.34-1.37) | 4815  1193.50  1.68 (1.60-1.76) | 22,345  396.26  1.44 (1.41-1.46) | 42,089  166.44  1.30 (1.28-1.32) | 39,639  246.85  1.32 (1.30-1.34) | 29,608  193.89  1.42 (1.39-1.44) | 61,053  201.15  1.36 (1.34-1.37) | 8194  839.43  1.10 (1.07-1.14) |
| 2012 | 67,476  219.81  1.35 (1.34-1.37) | 4972  1258.55  1.77 (1.69-1.85) | 21,468  402.37  1.46 (1.43-1.49) | 41,036  164.36  1.28 (1.26-1.30) | 38,621  245.46  1.31 (1.29-1.33) | 28,855  192.83  1.41 (1.39-1.44) | 59,267  199.63  1.35 (1.33-1.36) | 8206  813.05  1.07 (1.03-1.11) |
| 2013 | 66,642  222.56  1.37 (1.35-1.38) | 4709  1231.87  1.73 (1.66-1.82) | 21,465  409.08  1.48 (1.45-1.51) | 40,468  166.44  1.30 (1.28-1.32) | 37,993  247.50  1.32 (1.30-1.34) | 28,649  196.33  1.44 (1.41-1.46) | 57,927  200.27  1.35 (1.33-1.37) | 8715  855.81  1.13 (1.09-1.16) |
| 2014 | 62,294  213.59  1.31 (1.30-1.33) | 4540  1165.18  1.64 (1.57-1.72) | 20,258  394.37  1.43 (1.40-1.46) | 37,496  158.62  1.24 (1.22-1.25) | 35,686  238.85  1.27 (1.26-1.29) | 26,608  187.06  1.37 (1.35-1.40) | 54,362  193.09  1.30 (1.29-1.32) | 7932  784.60  1.03 (1.00-1.07) |
| 2015 | 53,901  188.69  1.16 (1.15-1.17) | 4473  1126.53  1.58 (1.51-1.66) | 17,651  338.54  1.23 (1.20-1.25) | 31,777  138.43  1.07 (1.06-1.10) | 30,802  210.45  1.12 (1.11-1.14) | 23,099  165.83  1.21 (1.19-1.24) | 46,929  170.33  1.15 (1.13-1.16) | 6972  688.44  0.91 (0.87-0.94) |
| 2016 | 58,103  204.58  1.26 (1.24-1.27) | 4994  1183.22  1.66 (1.59-1.74) | 20,202  369.02  1.34 (1.31-1.37) | 32,907  146.23  1.14 (1.12-1.16) | 33,095  227.01  1.21 (1.19-1.23) | 25,008  180.92  1.32 (1.30-1.35) | 51,030  186.39  1.26 (1.24-1.27) | 7073  691.29  0.91 (0.88-0.94) |
| Total | 1,096,069  194.31 | 72,871  967.71 | 346,172  335.33 | 676,955  149.34 | 633,895  219.21 | 462,103  168.11 | 962,825  175.78 | 133,173  816.42 |

Each cell contains (from top to bottom) (1) the number of events, (2) the incidence rate (per 1000 person-years), and (3) the incidence rate ratios (IRR) with 95% Poisson confidence intervals (CI) compared to the reference year 1999. Left-hand side of table: The totals of (1)-(3) are presented by calendar year. Right-hand side of table: For each calendar year (1)-(3) are presented by age group, gender, and presence of chronic disease.

**Text G in S1 File. Outpatient contacts.**

Reimbursements from private hospitals were only registered from 2002 onwards. The increase in the rate of outpatient contacts from 2001 to 2002 (most predominant in the neonatal age group) was partly explained by the introduction of registrations of radiological examinations (ICD-code Z01.6).

In the rate of outpatient contacts in the neonatal group seemed to have been tripled from 2004 to 2005 and after that point in time the new level was sustained. This finding was explained by the introduction of a neonatal screening for congenital hearing loss (ICD10-code Z13.5 (C)). Consequently, the results in the paper were obtained by excluding contacts with this diagnosis exclusively.

**Gender:** Overall boys had a 22 percent larger rate of outpatient contacts that that of girls. However, the rate increased more among girls with IRR_2016_ being 1.74 (1.71-1.77) for girls and 1.53 (1.51-1.55) for boys.

Table G in S1 File. Outpatient contacts among 0-5-year-old Danish children in the period 1999-2016.

|  | Events  Rate (per 1000)  IRR (95% CI)  vs. 1999 | **Age** | | | **Gender** | | **Chronic disease** | |
| --- | --- | --- | --- | --- | --- | --- | --- | --- |
| **Year** | **Total** | **0-28 days** | **28-365 days** | **1-5 years** | **Male** | **Female** | **No** | **Yes** |
| 1999 | 55,438  165.79  Ref. year for IRR | 4337  855.76 | 13,683  224.51 | 37,418  139.42 | 32,519  189.32 | 22,919  140.96 | 50,028  153.18 | 5410  692.34 |
| 2000 | 53,795  162.75  0.98 (0.97-0.99) | 6469  1266.24  1.48 (1.42-1.54) | 12,961  213.22  0.95 (0.93-0.97) | 34,365  129.85  0.93 (0.92-0.95) | 31,331  184.47  0.97 (0.96-0.99) | 22,464  139.78  0.99 (0.97-1.01) | 48,934  151.72  0.99 (0.98-1.00) | 4861  606.37  0.88 (0.85-0.91) |
| 2001 | 60,591  184.29  1.11 (1.10-1.12) | 7510  1496.36  1.75 (1.68-1.82) | 14,655  240.12  1.07 (1.04-1.09) | 38,426  146.25  1.05 (1.03-1.06) | 34,951  207.15  1.09 (1.08-1.11) | 25,640  160.18  1.14 (1.12-1.16) | 54,948  171.39  1.12 (1.11-1.13) | 5643  688.37  1.00 (0.96-1.04) |
| 2002 | 76,417  234.73  1.42 (1.40-1.43) | 12,456  2539.62  2.97 (2.87-3.08) | 17,248  290.63  1.29 (1.27-1.32) | 46,713  178.77  1.28 (1.26-1.30) | 43,623  261.37  1.38 (1.36-1.40) | 32,794  206.70  1.47 (1.44-1.49) | 69,903  220.32  1.44 (1.42-1.45) | 6514  786.19  1.14 (1.10-1.18) |
| 2003 | 74,736  231.25  1.39 (1.38-1.41) | 11,157  2255.88  2.64(2.55-2.73) | 17,773  302.43  1.35 (1.32-1.38) | 45,806  176.53  1.27 (1.25-1.28) | 42,654  257.42  1.36 (1.34-1.38) | 32,082  203.70  1.45 (1.42-1.47) | 67,827  215.52  1.41 (1.39-1.42) | 6909  814.36  1.18 (1.14-1.22) |
| 2004 | 78,003  242.63  1.46 (1.45-1.48) | 12,120  2458.31  2.87 (2.78-2.97) | 18,661  313.93  1.40 (1.37-1.43) | 47,222  183.67  1.32 (1.30-1.34) | 44,129  267.77  1.41 (1.39-1.43) | 33,874  216.20  1.53 (1.51-1.56) | 71,319  227.85  1.49 (1.47-1.50) | 6684  788.66  1.14 (1.10-1.19) |
| 2005 | 82,988  259.67  1.57 (1.55-1.58) | 17,597  3565.32  4.16 (4.03-4.31) | 19,188  323.78  1.44 (1.41-1.47) | 46,203  180.91  1.30 (1.28-1.32) | 46,778  285.55  1.51 (1.49-1.53) | 36,210  232.45  1.65 (1.62-1.68) | 76,299  245.32  1.60 (1.58-1.62) | 6689  779.62  1.13 (1.09-1.17) |
| 2006 | 87,454  275.33  1.66 (1.64-1.68) | 18,673  3749.24  4.38 (4.24-4.53) | 20,521  346.36  1.54 (1.51-1.58) | 48,260  190.45  1.37 (1.35-1.38) | 49,531  304.45  1.61 (1.59-1.63) | 37,923  244.76  1.74 (1.71-1.77) | 80,432  260.35  1.70 (1.68-1.72) | 7022  807.47  1.17 (1.13-1.21) |
| 2007 | 89,484  281.85  1.70 (1.68-1.72) | 21,543  4380.62  5.12 (4.96-5.29) | 20,819  351.26  1.56 (1.53-1.60) | 47,122  186.04  1.33 (1.32-1.35) | 49,936  306.89  1.62 (1.60-1.64) | 39,548  255.54  1.81 (1.78-1.84) | 82,606  267.68  1.75 (1.73-1.77) | 6878  774.34  1.12 (1.08-1.16) |
| 2008 | 96,360  302.60  1.83 (1.81-1.84) | 21,008  4216.47  4.93 (4.77-5.09) | 22,737  381.64  1.70 (1.66-1.74) | 52,615  207.25  1.49 (1.47-1.51) | 53,425  327.59  1.73 (1.71-1.75) | 42,935  276.37  1.96 (1.93-1.99) | 87,402  282.51  1.84 (1.82-1.86) | 8958  989.10  1.43 (1.39-1.48) |
| 2009 | 99,278  312.03  1.88 (1.86-1.90) | 21,630  4492.27  5.25 (5.08-5.42) | 23,558  398.95  1.78 (1.74-1.81) | 54,090  212.70  1.53 (1.51-1.55) | 55,613  340.95  1.80 (1.78-1.83) | 43,665  281.60  2.00 (1.97-2.03) | 90,846  294.09  1.92 (1.90-1.94) | 8432  910.47  1.32 (1.28-1.37) |
| 2010 | 100,075  315.36  1.90 (1.88-1.92) | 23,133  4742.01  5.54 (5.37-5.72) | 23,855  409.54  1.82 (1.79-1.86) | 53,087  208.84  1.50 (1.48-1.52) | 55,950  343.72  1.82 (1.79-1.84) | 44,125  285.50  2.03 (1.99-2.06) | 92,512  300.56  1.96 (1.94-1.98) | 7563  793.16  1.15 (1.11-1-19) |
| 2011 | 104,376  332.20  2.00 (1.98-2.02) | 23,503  5169.83  6.04 (5.85-6.24) | 25,493  449.69  2.00 (1.96-2.05) | 55,380  218.93  1.57 (1.55-1.59) | 58,590  363.81  1.92 (1.90-1.95) | 45,786  298.97  2.12 (2.09-2.16) | 96,136  315.84  2.06 (2.04-2.08) | 8240  839.61  1.22 (1.18-1.26) |
| 2012 | 104,326  338.83  2.04 (2.02-2.06) | 24,350  5482.38  6.41 (6.20-6.62) | 25,198  469.77  2.09 (2.05-2.14) | 54,778  219.27  1.57 (1.55-1.59) | 58,463  370.42  1.96 (1.93-1.98) | 45,863  305.61  2.17 (2.13-20) | 95,795  321.73  2.10 (2.08-2.12) | 8531  840.54  1.22 (1.18-1.26) |
| 2013 | 103,013  342.98  2.07 (2.05-2.09) | 22,507  5241.12  6.13 (5.93-6.33) | 25,977  492.51  2.19 (2.15-2.24) | 54,529  224.12  1.61 (1.59-1.63) | 57,244  371.73  1.96 (1.94-1.99) | 45,769  312.73  2.22 (2.18-2.25) | 94,787  326.73  2.13 (2.11-2.16) | 8226  803.57  1.17 (1.13-1.21) |
| 2014 | 98,003  334.99  2.02 (2.00-2.04) | 20,092  4605.78  5.38 (5.21-5.56) | 26,795  518.95  2.31 (2.26-2.36) | 51,116  216.08  1.55 (1.53-1.57) | 54,549  363.94  1.92 (1.90-1.95) | 43,454  304.57  2.16 (2.13-2.20) | 90,617  320.88  2.09 (2.07-2.12) | 7386  727.46  1.06 (1.02-109) |
| 2015 | 90,660  316.27  1.91 (1.89-1.93) | 19,383  4360.38  5.10 (4.93-5.27) | 24,507  467.34  2.08 (2.04-2.13) | 46,770  203.55  1.46 (1.44-1.48) | 49,993  340.37  1.80 (1.77-1.82) | 40,667  290.95  2.06 (2.03-2.10) | 83,657  302.57  1.97 (1.95-2.00) | 7003  688.96  1.00 (0.96-1.04) |
| 2016 | 76,378  268.08  1.62 (1.60-1.63) | 20,028  4262.95  4.98 (4.82-5.15) | 21,052  382.94  1.71 (1.67-1.74) | 35,298  156.72  1.12 (1.11-1.14) | 42,337  289.45  1.53 (1.51-1.55) | 34,041  245.54  1.74 (1.71-1.77) | 71,396  259.97  1.70 (1.68-1.72) | 4982  485.06  0.70 (0.68-0.73) |
| Total | 1,531,375  270.61 | 307,496  3564.17 | 374,681  361.28 | 849,198  187.22 | 861,616  296.92 | 669,759  242.90 | 1,405,444  255.76 | 125,931  767.37 |

Each cell contains (from top to bottom) (1) the number of events, (2) the incidence rate (per 1000 person-years), and (3) the incidence rate ratios (IRR) with 95% Poisson confidence intervals (CI) compared to the reference year 1999. Left-hand side of table: The totals of (1)-(3) are presented by calendar year. Right-hand side of table: For each calendar year (1)-(3) are presented by age group, gender, and presence of chronic disease.

**Text H in S1 File. Emergency contacts.**

In addition to the outcomes presented in the main article emergency contacts from The National Patient Register is presented in this supplement. The concept of emergency contacts was only defined in the register up to and including 2013. From 2014 onwards, the concept of *acute* outpatient contacts was introduced. These outpatient contacts were excluded.

The rate of emergency contacts gradually decreased over the years, IRR_2013_ = 0.75 (0.74-0.76), from 199.33 contacts per 1000 person-years in 1999 to 149.00 in 2013.

**Age:** The rate of emergency contacts was positively associated with age. The comparisons with the neonatal age group yielded IRR_28-365 days_ = 1.35 (1.31-1.40) and IRR_1-5 years_ = 2.95 (2.86-3.04). The decrease over time was larger among neonates and children aged 28-365 days with IRR_2013_ = 0.33 (0.27-0.41) and IRR_2013_ = 0.49 (0.47-0.51), respectively.

**Chronic disease:** The children with chronic disease had a higher rate of emergency contacts than the healthy children, IRR_chronic_ = 1.35 (1.34-1.37). The decrease over time was larger among children with chronic disease, IRR_2013_ = 0.66 (0.63-0.70) compared to IRR_2013_ = 0.75 (0.74-0.76) among healthy children.

**Gender:** The rate of emergency contacts was higher among boys than among girls, IRR_boys_=1.31 (1.31-1.32). The development over time was similar in the two groups.

Table H in S1 File. Emergency contacts among 0-5-year-old Danish children in the period 1999-2013.

|  | Events  Rate (per 1000)  IRR (95% CI)  vs. 1999 | **Age** | | | **Gender** | | **Chronic disease** | |
| --- | --- | --- | --- | --- | --- | --- | --- | --- |
| **Year** | **Total** | **0-28 days** | **28-365 days** | **1-5 years** | **Male** | **Female** | **No** | **Yes** |
| 1999 | 66,425  199.33  Ref. year for IRR | 433  100.27 | 7853  129.26 | 58,139  216.80 | 38,685  226.03 | 27,740  171.15 | 64,092  196.97 | 2333  297.57 |
| 2000 | 63,926  193.99  0.97 (0.96-0.98) | 441  100.99  1.00 (0.88-1.15) | 7053  116.32  0.90 (0.87-0.93) | 56,432  213.33  0.98 (0.97-1.00) | 36,913  218.04  0.96 (0.95-0.98) | 27,013  168.57  0.98 (0.97-1.00) | 61,799  192.20  0.98 (0.97-0.99) | 2127  265.69  0.89 (0.84-0.95) |
| 2001 | 63,879  194.79  0.98 (0.97-0.99) | 456  106.42  1.06 (0.93-1.21) | 7296  119.73  0.93 (0.90-0.96) | 56,127  213.64  0.99 (0.97-1.00) | 36,817  218.83  0.97 (0.95-0.98) | 27,062  169.44  0.99 (0.97-1.01) | 61,714  193.00  0.98 (0.97-0.99) | 2165  264.32  0.89 (0.84-0.94) |
| 2002 | 60,938  187.62  0.94 (0.93-0.95) | 403  96.07  0.96 (0.84-1.10) | 6258  105.63  0.82 (0.79-0.84) | 54,277  207.68  0.96 (0.95-0.97) | 35,369  212.48  0.94 (0.93-0.95) | 25,569  161.48  0.94 (0.93-0.96) | 58,807  185.80  0.94 (0.93-0.95) | 2131  257.37  0.86 (0.82-0.92) |
| 2003 | 56,357  174.76  0.88 (0.87-0.89) | 356  83.58  0.83 (0.72-0.96) | 5366  91.48  0.71 (0.68-0.73) | 50,635  195.07  0.90 (0.89-0.91) | 32,945  199.31  0.88 (0.87-0.89) | 23,412  148.93  0.87 (0.86-0.89) | 54,417  173.29  0.88 (0.87-0.89) | 1940  228.90  0.77 (0.72-0.82) |
| 2004 | 54,189  168.90  0.85 (0.84-0.86) | 312  72.87  0.73 (0.63-0.84) | 5090  85.77  0.66 (0.64-0.69) | 48,787  189.68  0.87 (0.86-0.89) | 31,477  191.42  0.85 (0.83-0.86) | 22,712  145.21  0.85 (0.83-0.86) | 52,234  167.22  0.85 (0.84-0.86) | 1955  230.95  0.77 (0.73-0.82) |
| 2005 | 53,006  166.17  0.83 (0.82-0.84) | 257  59.56  0.59 (0.51-0.69) | 4861  82.16  0.64 (0.61-0.66) | 47,888  187.43  0.86 (0.85-0.88) | 30,644  187.42  0.83 (0.82-0.84) | 22,362  143.82  0.84 (0.83-0.86) | 51,075  164.54  0.84 (0.83-0.85) | 1931  225.25  0.76 (0.71-0.80) |
| 2006 | 51,696  163.09  0.82 (0.81-0.83) | 236  54.09  0.54 (0.46-0.63) | 4708  79.65  0.62 (0.59-0.64) | 46,752  184.42  0.85 (0.84-0.86) | 30,090  185.33  0.82 (0.81-0.83) | 21,606  139.73  0.82 (0.80-0.83) | 49,806  161.55  0.82 (0.81-0.83) | 1890  217.43  0.73 (0.69-0.78) |
| 2007 | 52,462  165.59  0.83 (0.82- 0.84) | 210  48.67  0.49 (0.41-0.57) | 4725  79.91  0.62 (0.60-0.64) | 47,527  187.58  0.87 (0.85-0.88) | 30,437  187.46  0.83 (0.82-0.84) | 22,025  142.60  0.83 (0.82-0.85) | 50,490  163.96  0.83 (0.82-0.84) | 1972  222.15  0.75 (0.70-0.79) |
| 2008 | 52,252  164.42  0.82 (0.81- 0.83) | 243  55.24  0.55 (0.47-0.64) | 4502  75.75  0.59 (0.56-0.61) | 47,507  187.11  0.86 (0.85-0.87) | 30,290  186.16  0.82 (0.81-0.84) | 21,962  141.66  0.83 (0.81-0.84) | 50,344  163.09  0.83 (0.82-0.84) | 1908  210.92  0.71 (0.67-0.75) |
| 2009 | 56,075  176.61  0.89 (0.88-0.90) | 242  56.45  0.56 (0.48-0.66) | 4959  84.21  0.65 (0.63-0.68) | 50,874  200.02  0.92 (0.91-0.93) | 32,582  200.19  0.89 (0.87-0.90) | 23,493  151.80  0.88 (0.87-0.90) | 53,905  174.87  0.89 (0.88-0.90) | 2170  234.53  0.79 (0.74-0.84) |
| 2010 | 54,644  172.56  0.87 (0.86-0.88) | 193  44.27  0.44 (0.37-0.52) | 4672  80.44  0.62 (0.60-0.65) | 49,779  195.80  0.90 (0.89-0.91) | 31,612  194.61  0.86 (0.85-.087) | 23,032  149.32  0.87 (0.86-0.89) | 52,468  170.83  0.87 (0.86-0.88) | 2176  228.41  0.77 (0.72-0.81) |
| 2011 | 53,025  169.11  0.85 (0.84-0.86) | 165  40.62  0.41 (0.34-0.48) | 4193  74.22  0.57 (0.55-0.60) | 48,667  192.36  0.89 (0.88-0.90) | 30,706  191.04  0.85 (0.83-0.86) | 22,319  146.04  0.85 (0.84-0.87) | 50,816  167.30  0.85 (0.84-0.86) | 2209  225.29  0.76 (0.71-0.80) |
| 2012 | 48,663  158.38  0.79 (0.79-0.80) | 136  34.17  0.34 (0.28-0.41) | 3551  66.43  0.51 (0.49-0.53) | 44,976  180.04  0.83 (0.82-0.84) | 28,241  179.32  0.79 (0.78-0.81) | 20,422  136.36  0.80 (0.78-0.81) | 46,468  156.40  0.79 (0.78-0.80) | 2195  216.42  0.73 (0.69-0.77) |
| 2013 | 44,652  149.00  0.75 (0.74-0.76) | 129  33.51  0.33 (0.27-0.41) | 3357  63.86  0.49 (0.47-0.51) | 41,166  169.22  0.78 (0.77-0.79) | 25,978  169.07  0.75 (0.74-0.76) | 18,674  127.87  0.75 (0.73-0.76) | 42,631  147.28  0.75 (0.74-0.76) | 2021  197.54  0.66 (0.63-0.70) |
| Total | 832,189  173.95 | 4212  66.17 | 78,444  89.56 | 749,533  194.96 | 482,786  196.83 | 349,403  149.88 | 801,066  172.25 | 31,123  233.30 |

Each cell contains (from top to bottom) (1) the number of events, (2) the incidence rate (per 1000 person-years), and (3) the incidence rate ratios (IRR) with 95% Poisson confidence intervals (CI) compared to the reference year 1999. Left-hand side of table: The totals of (1)-(3) are presented by calendar year. Right-hand side of table: For each calendar year (1)-(3) are presented by age group, gender, and presence of chronic disease.

**Text I in S1 File. General practitioner contacts.**

**Gender:** Overall the rate was 8 percent larger among boys, IRR_boys_ = 1.08 (1.08-1.08), and the development over time was similar in the two groups.

**Table I in S1 File.** **General practitioner contacts among 0-5-year-old Danish children in the period 1999-2016.**

|  | Events  Rate (per 1000)  IRR (95% CI) vs. 1999 | **Age** | | | **Gender** | | **Chronic disease** | |
| --- | --- | --- | --- | --- | --- | --- | --- | --- |
| **Year** | **Total** | **0-28 days** | **28-365 days** | **1-5 years** | **Male** | **Female** | **No** | **Yes** |
| 1999 | 2,617,750  7820.97  Ref. year for IRR | 13,220  2645.37 | 639,837  10,511.69 | 1,964,693  7298.18 | 1,391,142  8075.20 | 1,226,608  7522.37 | 2,539,714  7755.45 | 78,036  9928.20 |
| 2000 | 2,670,329  8055.60  1.03 (1.03-1.03) | 23,248  4550.04  1.74 (1.71-1.78) | 672,707  11,045.05  1.05 (1.05-1.06) | 1,974,374  7437.22  1.02 (1.02-1.02) | 1,425,390  8369.52  1.04 (1.03-1.04) | 1,244,939  7723.91  1.03 (1.02-1.03) | 2,587,262  7998.83  1.03 (1.03-1.03) | 83,067  10,341.88  1.04 (1.03-1.05) |
| 2001 | 2,855,212  8658.85  1.11 (1.11-1.11) | 29,123  5802.55  2.23 (2.18-2.27) | 709,063  11,597.85  1.11 (1.10-1.11) | 2,117,026  8031.56  1.10 (1.10-1.10) | 1,522,940  9000.90  1.11 (1.11-1.12) | 1,332,272  8298.37  1.10 (1.10-1.11) | 2,766,784  8605.19  1.11 (1.11-1.11) | 88,428  10,758.01  1.08 (1.07-1.09) |
| 2002 | 2,853,293  8741.53  1.12 (1.12-1.12) | 35,208  7178.05  2.75 (2.70-2.81) | 688,792  11,589.47  1.11 (1.10-1.11) | 2,129,293  8124.93  1.11 (1.11-1.12) | 1,518,865  9076.69  1.12 (1.12-1.13) | 1,334,428  8388.95  1.12 (1.11-1.12) | 2,764,228  8689.75  1.12 (1.12-1.12) | 89,065  10,724.92  1.08 (1.07-1.09) |
| 2003 | 2,853,480  8808.23  1.13 (1.13-1.13) | 41,750  8441.24  3.24 (3.17-3.30) | 679,867  11,551.62  1.10 (1.10-1.11) | 2,131,863  8194.58  1.12 (1.12-1.13) | 1,519,932  9151.28  1.13 (1.13-1.14) | 1,333,548  8447.32  1.12 (1.12-1.13) | 2,762,506  8757.21  1.13 (1.13-1.13) | 90,974  10,701.66  1.08 (1.07-1.09) |
| 2004 | 2,755,395  8550.94  1.10 (1.09-1.10) | 45,721  9272.69  3.56 (3.49-3.63) | 675,707  11,348.95  1.08 (1.08-1.09) | 2,033,967  7890.84  1.08 (1.08-1.08) | 1,468,615  8889.76  1.10 (1.10-1.10) | 1,286,780  8194.48  1.09 (1.09-1.09) | 2,669,479  8508.38  1.10 (1.10-1.10) | 85,916  10,124.44  1.02 (1.01-1.03) |
| 2005 | 2,728,957  8516.15  1.09 (1.09-1.09) | 49,275  9982.34  3.83 (3.76-3.90) | 664,377  11,187.08  1.07 (1.06-1.07) | 2,015,305  7868.57  1.08 (1.08-1.08) | 1,452,347  8841.47  1.09 (1.09-1.10) | 1,276,610  8173.99  1.09 (1.08-1.09) | 2,641,542  8470.68  1.09 (1.09-1.09) | 87,415  10,165.25  1.02 (1.01-1.03) |
| 2006 | 2,755,558  8649.01  1.11 (1.11-1.11) | 50,012  10,040.27  3.85 (3.78-3.92) | 678,282  11,423.65  1.09 (1.09-1.09) | 2,027,264  7973.77  1.09 (1.09-1.09) | 1,464,608  8975.20  1.11 (1.11-1.11) | 1,290,950  8306.51  1.10 (1.10-1.11) | 2,665,998  8603.29  1.11 (1.11-1.11) | 89,560  10,274.14  1.03 (1.02-1.04) |
| 2007 | 2,870,466  9012.88  1.15 (1.15-1.16) | 54,062  10,992.60  4.22 (4.14-4.30) | 705,475  11,882.30  1.13 (1.13-1.14) | 2,110,929  8304.38  1.14 (1.14-1.14) | 1,522,642  9327.69  1.16 (1.15-1.16) | 1,347,824  8681.87  1.15 (1.15-1.16) | 2,776,098  8967.47  1.16 (1.15-1.16) | 94,368  10,590.48  1.07 (1.06-1.08) |
| 2008 | 2,730,766  8550.16  1.10 (1.09-1.10) | 61,601  12,362.16  4.74 (4.65-4.83) | 687,402  11,517.85  1.10 (1.10-1.10) | 1,981,763  7780.25  1.07 (1.06-1.07) | 1,450,701  8868.60  1.10 (1.10-1.10) | 1,280,065  8215.84  1.09 (1.09-1.09) | 2,641,469  8512.49  1.10 (1.10-1.10) | 89,297  9838.23  0.99 (0.98-1.00) |
| 2009 | 2,662,869  8345.91  1.07 (1.07-1.07) | 52,408  10,883.61  4.17 (4.09-4.25) | 663,502  11,216.98  1.07 (1.07-1.07) | 1,946,959  7632.26  1.05 (1.04-1.05) | 1,418,502  8670.34  1.07 (1.07-1.08) | 1,244,367  8004.47  1.06 (1.06-1.07) | 2,569,695  8295.10  1.07 (1.07-1.07) | 93,174  10,042.36  1.01 (1.00-1.02) |
| 2010 | 2,736,760  8603.02  1.10 (1.10-1.10) | 42,245  8658.81  3.32 (3.26-3.39) | 673,879  11,549.95  1.10 (1.10-1.11) | 2,020,636  7927.40  1.09 (1.08-1.09) | 1,451,466  8893.90  1.10 (1.10-1.10) | 1,285,294  8296.60  1.10 (1.10-1.11) | 2,638,942  8552.29  1.10 (1.10-1.10) | 97,818  10,242.08  1.03 (1.02-1.04) |
| 2011 | 2,708,082  8597.54  1.10 (1.10-1.10) | 42,683  9387.96  3.60 (3.53-3.67) | 652,765  11,492.85  1.10 (1.09-1.10) | 2,012,634  7935.03  1.09 (1.09-1.09) | 1,432,267  8871.48  1.10 (1.10-1.10) | 1,275,815  8309.50  1.10 (1.10-1.11) | 2,607,523  8544.89  1.10 (1.10-1.10) | 100,559  10,232.54  1.03 (1.02-1.04) |
| 2012 | 2,495,566  8083.67  1.04 (1.03-1.04) | 42,096  9476.87  3.63 (3.56-3.71) | 605,691  11,272.53  1.08 (1.07-1.08) | 1,847,779  7375.08  1.01 (1.01-1.01) | 1,320,164  8343.66  1.03 (1.03-1.04) | 1,175,402  7810.32  1.04 (1.04-1.04) | 2,398,600  8034.27  1.04 (1.03-1.04) | 96,966  9533.55  0.96 (0.95-0.97) |
| 2013 | 2,322,740  7713.41  0.99 (0.99-0.99) | 40,313  9387.29  3.60 (3.53-3.67) | 570,798  10,806.34  1.03 (1.03-1.03) | 1,711,629  7014.44  0.96 (0.96-0.96) | 1,229,146  7961.85  0.99 (0.98-0.99) | 1,093,594  7452.05  0.99 (0.99-0.99) | 2,230,360  7667.79  0.99 (0.99-0.99) | 92,380  9007.20  0.91 (0.90-0.92) |
| 2014 | 2,134,403  7278.03  0.93 (0.93-0.93) | 38,542  8835.06  3.39 (3.32-3.46) | 520,961  10,076.71  0.96 (0.96-0.97) | 1,574,900  6639.41  0.91 (0.91-0.91) | 1,127,106  7502.34  0.93 (0.93-0.93) | 1,007,297  7042.43  0.94 (0.93-0.94) | 2,048,725  7236.86  0.93 (0.93-0.93) | 85,678  8423.96  0.85 (0.84-0.86) |
| 2015 | 1,934,338  6735.55  0.86 (0.86-0.86) | 37,614  8461.17  3.24 (3.18-3.31) | 484,702  9235.41  0.88 (0.88-0.88) | 1,412,022  6132.43  0.84 (0.84-0.84) | 1,022,303  6947.24  0.86 (0.86-0.86) | 912,035  6513.09  0.87 (0.86-0.87) | 1,853,605  6691.55  0.86 (0.86-0.86) | 80,733  7933.14  0.80 (0.79-0.81) |
| 2016 | 1,973,232  7116.44  0.91 (0.91-0.91) | 40,790  8900.81  3.41 (3.35-3.48) | 523,651  9798.52  0.94 (0.93-0.94) | 1,408,791  6425.40  0.88 (0.88-0.88) | 1,043,814  7332.86  0.91 (0.91-0.91) | 929,418  6888.13  0.92 (0.91-0.92) | 1,890,231  7072.95  0.91 (0.91-0.91) | 83,001  8275.28  0.83 (0.83-0.84) |
| Total | 46,659,196  8235.23 | 739,911  8587.08 | 11,497,458  11,084.89 | 34,421,827  7577.96 | 24,781,950  8530.04 | 21,877,246  7924.96 | 45,052,761  8188.97 | 1,606,435  9785.33 |

Each cell contains (from top to bottom) (1) the number of events, (2) the incidence rate (per 1000 person-years), and (3) the incidence rate ratios (IRR) with 95% Poisson confidence intervals (CI) compared to the reference year 1999. Left-hand side of table: The totals of (1)-(3) are presented by calendar year. Right-hand side of table: For each calendar year (1)-(3) are presented by age group, gender, and presence of chronic disease.

**Text J in S1 File. Medical specialist contacts.**

**Gender:** Overall the rate of specialist contacts was 29 percent larger among boys than among girls, IRR_boys_ = 1.29 (1.29-1.29). The development over time was similar in the two groups.

**Table J in S1 File.** **Medical specialist contacts among 0-5-year-old Danish children in the period 1999-2016.**

|  | Events  Rate (per 1000)  IRR (95% CI) vs. 1999 | **Age** | | | **Gender** | | **Chronic disease** | |
| --- | --- | --- | --- | --- | --- | --- | --- | --- |
| **Year** | **Total** | **0-28 days** | **28-365 days** | **1-5 years** | **Male** | **Female** | **No** | **Yes** |
| 1999 | 521,225  1554.34  Ref. year for IRR | 216  42.61 | 63,986  1047.88 | 457,023  1697.69 | 298,817  1734.55 | 222,408  1363.95 | 503,219  1536.66 | 18,006  2290.83 |
| 2000 | 553,869  1670.86  1.07 (1.07-1.08) | 316  61.84  1.45 (1.22-1.73) | 71,669  1176.72  1.12 (1.11-1.13) | 481,884  1815.19  1.07 (1.06-1.07) | 319,498  1876.01  1.08 (1.08-1.09) | 234,371  1454.09  1.07 (1.06-1.07) | 533,945  1650.75  1.07 (1.07-1.08) | 19,924  2480.54  1.08 (1.06-1.10) |
| 2001 | 579,907  1758.65  1.13 (1.13-1.14) | 300  59.77  1.40 (1.18-1.67) | 73,380  1200.24  1.15 (1.13-1.16) | 506,227  1920.52  1.13 (1.13-1.14) | 335,465  1982.67  1.14 (1.14-1.15) | 244,442  1522.56  1.12 (1.11-1.13) | 558,333  1736.51  1.13 (1.13-1.13) | 21,574  2624.66  1.15 (1.12-1.17) |
| 2002 | 588,180  1801.98  1.16 (1.16-1.16) | 343  69.92  1.64 (1.38-1.94) | 72,617  1221.83  1.17 (1.15-1.18) | 515,220  1965.97  1.16 (1.15-1.16) | 340,704  2036.03  1.17 (1.17-1.18) | 247,476  1555.77  1.14 (1.13-1.15) | 567,019  1782.50  1.16 (1.16-1.16) | 21,161  2548.14  1.11 (1.09-1.13) |
| 2003 | 600,804  1854.58  1.19 (1.19-1.20) | 443  89.56  2.10 (1.78-2.47) | 72,401  1230.15  1.17 (1.16-1.19) | 527,960  2029.40  1.20 (1.19-1.20) | 347,081  2089.72  1.20 (1.20-1.21) | 253,723  1607.19  1.18 (1.17-1.9) | 577,440  1830.49  1.19 (1.19-1.20) | 23,364  2748.40  1.20 (1.18-1.22) |
| 2004 | 635,260  1971.42  1.27 (1.26-1.27) | 490  99.37  2.33 (1.98-2.73) | 77,138  1295.57  1.24 (1.22-1.25) | 557,632  2163.34  1.27 (1.27-1.28) | 365,605  2213.06  1.28 (1.27-1.28) | 269,655  1717.20  1.26 (1.25-1.27) | 610,784  1946.73  1.27 (1.26-1.27) | 24,476  2884.28  1.26 (1.24-1.28) |
| 2005 | 607,552  1895.95  1.22 (1.22-1.22) | 593  120.13  2.81 (2.41-3.29) | 72,903  1227.55  1.17 (1.16-1.18) | 534,056  2085.16  1.23 (1.22-1.23) | 348,963  2124.38  1.22 (1.22-1.23) | 258,589  1655.69  1.21 (1.21-1.22) | 583,928  1872.48  1.22 (1.21-1.22) | 23,624  2747.17  1.20 (1.18-1.22) |
| 2006 | 611,369  1918.92  1.23 (1.23-1.24) | 549  110.21  2.58 (2.20-3.02) | 73,016  1229.73  1.17 (1.16-1.19) | 537,804  2115.31  1.25 (1.24-1.25) | 352,316  2159.01  1.24 (1.24-1.25) | 259,053  1666.83  1.22 (1.22-1.23) | 587,526  1895.96  1.23 (1.23-1.24) | 23,843  2735.22  1.19 (1.17-1.22) |
| 2007 | 639,617  2008.29  1.29 (1.29-1.30) | 594  120.77  2.83 (2.42-3.31) | 74,541  1255.48  1.20 (1.19-1.21) | 564,482  2220.65  1.31 (1.30-1.31) | 365,111  2236.66  1.29 (1.28-1.30) | 274,506  1768.17  1.30 (1.29-1.30) | 614,346  1984.47  1.29 (1.29-1.30) | 25,271  2836.04  1.24 (1.21-1.26) |
| 2008 | 646,439  2024.01  1.30 (1.30-1.31) | 730  146.49  3.43 (2.95-4.00) | 74,071  1241.07  1.18 (1.17-1.20) | 571,638  2244.19  1.32 (1.32-1.33) | 372,657  2278.17  1.31 (1.31-1.32) | 273,782  1757.17  1.29 (1.28-1.30) | 620,951  2001.07  1.30 (1.30-1.31) | 25,488  2808.12  1.23 (1.20-1.25) |
| 2009 | 656,974  2059.05  1.32 (1.32-1.33) | 830  172.36  4.04 (3.48-4.69) | 80,920  1368.00  1.31 (1.29-1.32) | 575,224  2254.90  1.33 (1.32-1.33) | 378,484  2313.41  1.33 (1.33-1.34) | 278,490  1791.37  1.31 (1.31-1.32) | 631,736  2039.25  1.33 (1.32-1.33) | 25,238  2720.16  1.19 (1.16-121) |
| 2010 | 713,123  2241.68  1.44 (1.44-1.45) | 1049  215.00  5.04 (4.35-5.84) | 86,327  1479.58  1.41 (1.40-1.43) | 625,747  2454.92  1.45 (1.44-1.45) | 410,845  2517.46  1.45 (1.44-1.46) | 302,278  1951.17  1.43 (1.42-1.44) | 683,942  2216.50  1.44 (1.44-1.45) | 29,181  3055.41  1.33 (1.31-1.36) |
| 2011 | 711,728  2259.55  1.45 (1.45-1.46) | 1075  236.44  5.54 (4.79-6.42) | 84,341  1484.94  1.42 (1.40-1.43) | 626,312  2469.27  1.45 (1.45-1.46) | 408,299  2529.00  1.46 (1.45-1.46) | 303,429  1976.22  1.45 (1.44-1.46) | 680,946  2231.44  1.45 (1.45-1.46) | 30,782  3132.27  1.37 (1.34-1.39) |
| 2012 | 678,920  2199.15  1.41 (1.41-1.42) | 1179  265.42  6.22 (5.38-7.20) | 77,953  1450.80  1.38 (1.37-1.40) | 599,788  2393.92  1.41 (1.40-1.42) | 389,337  2460.67  1.42 (1.41-1.43) | 289,583  1924.20  1.41 (1.40-1.42) | 647,914  2170.21  1.41 (1.41-1.42) | 31,006  3048.46  1.33 (1.31-1.36) |
| 2013 | 645,514  2143.64  1.38 (1.37-1.38) | 1258  292.94  6.87 (5.95-7.94) | 78,623  1488.52  1.42 (1.41-1.44) | 565,633  2318.02  1.37 (1.36-1.37) | 371,237  2404.72  1.39 (1.38-1.39) | 274,277  1869.00  1.37 (1.36-1.38) | 614,928  2114.07  1.38 (1.37-1.38) | 30,586  2982.18  1.30 (1.28-1.33) |
| 2014 | 637,512  2173.85  1.40 (1.39-1.40) | 1591  364.71  8.55 (7.42-9.86) | 80,344  1554.10  1.48 (1.47-1.50) | 555,577  2342.20  1.38 (1.37-1.39) | 365,804  2434.92  1.40 (1.40-1.41) | 271,708  1899.64  1.39 (1.38-1.40) | 607,222  2144.96  1.40 (1.39-1.40) | 30,290  2978.15  1.30 (1.28-1.32) |
| 2015 | 617,079  2148.78  1.38 (1.38-1.39) | 1835  412.80  9.68 (8.41-11.15) | 79,590  1516.51  1.45 (1.43-1.46) | 535,654  2326.41  1.37 (1.36-1.38) | 355,556  2416.28  1.39 (1.39-1.40) | 261,52  1867.66  1.37 (1.36-1.38) | 586,627  2117.78  1.38 (1.37-1.38) | 30,452  2992.33  1.31 (1.28-1.33) |
| 2016 | 615,383  2219.50  1.43 (1.42-1.43) | 2070  451.77  10.60 (9.21-12.19) | 86,778  1624.00  1.55 (1.53-1.57) | 526,535  2401.58  1.41 (1.41-1.42) | 355,838  2499.94  1.44 (1.43-1.45) | 259,545  1923.65  1.41 (1.40-1.42) | 583,332  2182.87  1.42 (1.42-1.43) | 32,051  3195.56  1.39 (1.37-1.42) |
| Total | 11,260,455  1987.44 | 15,461  179.43 | 1,380,598  1331.06 | 9,864,396  2171.61 | 6,481,617  2231.00 | 4,778,838  1731.11 | 10,794,138  1961.98 | 466,317  2840.49 |

Each cell contains (from top to bottom) (1) the number of events, (2) the incidence rate (per 1000 person-years), and (3) the incidence rate ratios (IRR) with 95% Poisson confidence intervals (CI) compared to the reference year 1999. Left-hand side of table: The totals of (1)-(3) are presented by calendar year. Right-hand side of table: For each calendar year (1)-(3) are presented by age group, gender, and presence of chronic disease.

**Text K in S1 File. Medication.**

**Gender:** Overall boys had a 16 percent higher prescription rate than girls, IRR_2016_ = 1.16 (1.15-1.17). The development over time was similar in the two groups.

Table K in S1 File. Prescribed medication use among 0-5-year-old Danish children in the period 1999-2016.

|  | Events  Rate (per 1000)  IRR (95% CI)  vs. 1999 | **Age** | | | **Gender** | | **Chronic disease** | |
| --- | --- | --- | --- | --- | --- | --- | --- | --- |
| **Year** | **Total** | **0-28 days** | **28-365 days** | **1-5 years** | **Male** | **Female** | **No** | **Yes** |
| 1999 | 824,167  2457.75  Ref. year for IRR | 2521  497.34 | 176,640  2892.79 | 645,009  2396.00 | 453,754  2633.92 | 370,416  2271.64 | 783,658  2393.03 | 40,512  5154.19 |
| 2000 | 840,987  2537.01  1.03 (1.03-1.04) | 4548  890.12  1.79 (1.70-1.88) | 182,850  3002.18  1.04 (1.03-1.04) | 653,589  2461.99  1.03 (1.02-1.03) | 464,285  2726.16  1.04 (1.03-1.04) | 376,699  2337.14  1.03 (1.02-1.03) | 799,175  2470.74  1.03 (1.03-1.04) | 41,809  5205.24  1.01 (1.00-1.02) |
| 2001 | 867,468  2630.73  1.07 (1.07-1.07) | 6272  1249.65  2.51 (2.40-2.63) | 188,248  3079.11  1.06 (1.06-1.07) | 672,948  2553.04  1.07 (1.06-1.07) | 479,537  2834.17  1.08 (1.07-1.08) | 387,934  2416.34  1.06 (1.06-1.07) | 823,932  2562.58  1.07 (1.07-1.07) | 43,539  5296.88  1.03 (1.01-1.04) |
| 2002 | 881,506  2700.64  1.10 (1.10-1.10) | 6787  1383.71  2.78 (2.66-2.91) | 177,822  2992.00  1.03 (1.03-1.04) | 696,897  2659.21  1.11 (1.11-1.11) | 486,532  2907.50  1.10 (1.10-1.11) | 394,973  2483.02  1.09 (1.09-1.10) | 837,710  2633.46  1.10 (1.10-1.10) | 43,795  5273.65  1.02 (1.01-1.04) |
| 2003 | 834,019  2574.48  1.05 (1.04-1.05) | 7561  1528.70  3.07 (2.94-3.22) | 162,935  2768.40  0.96 (0.95-0.96) | 663,523  2550.49  1.06 (1.06-1.07) | 460,941  2775.26  1.05 (1.05-1.06) | 373,078  2363.24  1.04 (1.04-1.05) | 791,151  2507.9  1.05 (1.04-1.05) | 42,868  5042.74  0.98 (0.97-0.99) |
| 2004 | 865,985  2687.44  1.09 (1.09-1.10) | 8030  1628.54  3.27 (3.13-3.42) | 171,886  2886.92  1.00 (0.99-1.00) | 686,069  2661.61  1.11 (1.11-1.11) | 476,730  2885.73  1.10 (1.09-1.10) | 389,253  2478.83  1.09 (1.09-1.10) | 822,563  2621.73  1.10 (1.09-1.10) | 43,420  5116.66  0.99 (0.98-1.01) |
| 2005 | 812,008  2533.99  1.03 (1.03-1.03) | 8027  1626.15  3.27 (3.13-3.42) | 159,838  2691.39  0.93 (0.92-0.94) | 644,143  2514.99  1.05 (1.05-1.05) | 447,531  2724.44  1.03 (1.03-1.04) | 364,475  2333.66  1.03 (1.02-1.03) | 768,938  2465.75  1.03 (1.03-1.03) | 43,068  5008.26  0.97 (0.96-0.98) |
| 2006 | 845,752  2654.59  1.08 (1.08-1.08) | 8045  1615.09  3.25 (3.11-3.40) | 160,835  2708.79  0.94 (0.93-0.94) | 676,872  2662.29  1.11 (1.11-1.11) | 467,615  2865.57  1.09 (1.08-1.09) | 378,135  2433.05  1.07 (1.07-1.08) | 800,773  2584.12  1.08 (1.08-1.08) | 44,977  5159.67  1.00 (0.99-1.01) |
| 2007 | 857,468  2692.32  1.10 (1.09-1.10) | 7633  1552.01  3.12 (2.98-3.26) | 156,701  2639.32  0.91 (0.91-0.92) | 693,134  2726.78  1.14 (1.13-1.14) | 473,265  2899.22  1.10 (1.10-1.11) | 384,208  2474.80  1.09 (1.08-1.09) | 810,728  2618.83  1.09 (1.09-1.10) | 46,745  5245.97  1.02 (1.00-1.03) |
| 2008 | 807,951  2529.71  1.03 (1.03-1.03) | 8115  1628.51  3.27 (3.13-3.42) | 148,946  2495.61  0.86 (0.86-0.87) | 650,890  2555.33  1.07 (1.06-1.07) | 445,579  2723.97  1.03 (1.03-1.04) | 362,371  2325.76  1.02 (1.02-1.03) | 763,507  2460.48  1.03 (1.02-1.03) | 44,443  4896.47  0.95 (0.94-0.96) |
| 2009 | 779,281  2442.38  0.99 (0.99-1.00) | 7677  1594.26  3.21 (3.06-3.35) | 148,139  2504.41  0.87 (0.86-0.87) | 623,465  2444.02  1.02 (1.02-1.02) | 431,355  2636.58  1.00 (1.00-1.01) | 347,927  2238.02  0.99 (0.98-0.99) | 734,921  2372.33  0.99 (0.99-0.99) | 44,361  4781.25  0.93 (0.92-0.94) |
| 2010 | 873,214  2744.93  1.12 (1.11-1.12) | 8080  1656.13  3.33 (3.18-3.48) | 160,786  2755.76  0.95 (0.95-0.96) | 704,348  2763.30  1.15 (1.15-1.16) | 481,825  2952.40  1.12 (1.12-1.13) | 391,390  2526.38  1.11 (1.11-1.12) | 824,206  2671.06  1.12 (1.11-1.12) | 49,009  5131.51  1.00 (0.98-1.01) |
| 2011 | 826,734  2624.67  1.07 (1.06-1.07) | 7784  1712.06  3.44 (3.29-3.60) | 149,560  2633.20  0.91 (0.90-0.92) | 669,390  2639.12  1.10 (1.10-1.11) | 452,767  2804.45  1.06 (1.06-1.07) | 373,967  2435.64  1.07 (1.07-1.08) | 775,653  2541.80  1.06 (1.06-1.07) | 51,081  5197.82  1.01 (1.00-1.02) |
| 2012 | 744,164  2410.48  0.98 (0.98-0.98) | 7109  1600.39  3.22 (3.08-3.37) | 129,985  2419.14  0.84 (0.83-0.84) | 607,070  2422.99  1.01 (1.01-1.01) | 407,485  2575.37  0.98 (0.97-0.98) | 336,680  2237.14  0.98 (0.98-0.99) | 693,239  2322.02  0.97 (0.97-0.97) | 50,926  5006.97  0.97 (0.96-0.98) |
| 2013 | 669,669  2223.84  0.90 (0.90-0.91) | 7521  1751.34  3.52 (3.37-3.68) | 121,755  2305.04  0.80 (0.79-0.80) | 540,393  2214.58  0.92 (0.92-0.93) | 367,595  2381.10  0.90 (0.90-0.91) | 302,074  2058.40  0.91 (0.90-0.91) | 621,469  2136.54  0.89 (0.89-0.90) | 48,200  4699.58  0.91 (0.90-0.92) |
| 2014 | 653,936  2229.82  0.91 (0.90-0.91) | 8210  1881.93  3.78 (3.62-3.96) | 117,583  2274.35  0.79 (0.78-0.79) | 528,143  2226.51  0.93 (0.93-0.93) | 357,925  2382.44  0.90 (0.90-0.91) | 296,011  2069.52  0.91 (0.91-0.92) | 606,390  2141.98  0.90 (0.89-0.90) | 47,546  4674.78  0.91 (0.90-0.92) |
| 2015 | 578,965  2015.98  0.82 (0.82-0.82) | 6625  1490.28  3.00 (2.86-3.14) | 105,445  2009.03  0.69 (0.69-0.70) | 466,896  2027.71  0.85 (0.84-0.85) | 317,433  2157.14  0.82 (0.82-0.82) | 261,532  1867.64  0.82 (0.82-0.83) | 534,970  1931.21  0.81 (0.80-0.81) | 43,995  4323.12  0.84 (0.83-0.85) |
| 2016 | 574,944  2016.49  0.82 (0.82-0.82) | 6571  1398.63  2.81 (2.69-2.94) | 109,220  1985.93  0.69 (0.68-0.69) | 459,153  2036.82  0.85 (0.85-0.85) | 316,151  2159.85  0.82 (0.82-0.82) | 258,793  1865.24  0.82 (0.82-0.83) | 530,499  1930.164  0.81 (0.80-0.81) | 44,445  4325.70  0.84 (0.83-0.85) |
| Total | 14,138,221  2491.90 | 127,116  1473.27 | 2,729,176  2627.28 | 11,281,929  2480.30 | 7,788,305  2677.05 | 6,349,916  2297.04 | 13,323,482  2418.38 | 814,739  4955.47 |

Each cell contains (from top to bottom) (1) the number of events, (2) the incidence rate (per 1000 person-years), and (3) the incidence rate ratios (IRR) with 95% Poisson confidence intervals (CI) compared to the reference year 1999. Left-hand side of table: The totals of (1)-(3) are presented by calendar year. Right-hand side of table: For each calendar year (1)-(3) are presented by age group, gender, and presence of chronic disease.

**Table L in S1 File. Age at first discharge from the hospital to the home after birth - by calendar year**

| **Year** | **Mean age (in days) at first discharge** | **Median age (in days) at first discharge** |
| --- | --- | --- |
| 1999 | 9.56 | 3 |
| 2000 | 9.59 | 3 |
| 2001 | 8.96 | 3 |
| 2002 | 7.82 | 3 |
| 2003 | 8.62 | 3 |
| 2004 | 8.07 | 3 |
| 2005 | 7.93 | 2 |
| 2006 | 8.95 | 2 |
| 2007 | 9.27 | 2 |
| 2008 | 8.91 | 2 |
| 2009 | 8.72 | 2 |
| 2010 | 8.03 | 2 |
| 2011 | 8.64 | 2 |
| 2012 | 7.41 | 2 |
| 2013 | 6.42 | 2 |
| 2014 | 5.33 | 2 |
| 2015 | 4.11 | 2 |
| 2016 | 3.17 | 2 |

**Appendix A in S1 File. Supplementary references.**

1. The Capital Region of Denmark. Medical Helpline 1813 [Internet]. [cited 2018 Aug 30]. Available from: https://www.regionh.dk/english/Healthcare-Services/Emergency-Medical-Services/Pages/Medical-Helpline-1813.aspx

2. Statistics Denmark. StatBank Denmark [Internet]. 2018 [cited 2018 Jul 13]. Available from: http://www.statistikbanken.dk/10188
